# Quantifying the effects of sleep on sensor-derived variables from upper limb accelerometry in people with and without upper limb impairment

**DOI:** 10.1186/s12984-024-01384-z

**Published:** 2024-05-28

**Authors:** Allison E. Miller, Catherine E. Lang, Marghuretta D. Bland, Keith R. Lohse

**Affiliations:** 1 Program in Physical Therapy, Washington University School of Medicine, 4444 Forest Park Avenue, MSC: 8502-66-1101, St. Louis, MO 63018 USA; 2 Program in Occupational Therapy, Washington University School of Medicine, St. Louis, MO 63018 USA; 3 Department of Neurology, Washington University School of Medicine, St. Louis, MO 63018 USA

**Keywords:** Stroke, Sleep, Wearable sensors, Accelerometry, Breast cancer, Multiple sclerosis, Fracture

## Abstract

**Background:**

Despite the promise of wearable sensors for both rehabilitation research and clinical care, these technologies pose significant burden on data collectors and analysts. Investigations of factors that may influence the wearable sensor data processing pipeline are needed to support continued use of these technologies in rehabilitation research and integration into clinical care settings. The purpose of this study was to investigate the effect of one such factor, sleep, on sensor-derived variables from upper limb accelerometry in people with and without upper limb impairment and across a two-day wearing period.

**Methods:**

This was a secondary analysis of data collected during a prospective, longitudinal cohort study (*n* = 127 individuals, 62 with upper limb impairment and 65 without). Participants wore a wearable sensor on each wrist for 48 h. Five upper limb sensor variables were calculated over the full wear period (sleep included) and with sleep time removed (sleep excluded): preferred time, non-preferred time, use ratio, non-preferred magnitude and its standard deviation. Linear mixed effects regression was used to quantify the effect of sleep on each sensor variable and determine if the effect differed between people with and without upper limb impairment and across a two-day wearing period.

**Results:**

There were significant differences between sleep included and excluded for the variables preferred time (*p* < 0.001), non-preferred time (*p* < 0.001), and non-preferred magnitude standard deviation (*p* = 0.001). The effect of sleep was significantly different between people with and without upper limb impairment for one variable, non-preferred magnitude (*p* = 0.02). The effect of sleep was not substantially different across wearing days for any of the variables.

**Conclusions:**

Overall, the effects of sleep on sensor-derived variables of upper limb accelerometry are small, similar between people with and without upper limb impairment and across a two-day wearing period, and can likely be ignored in most contexts. Ignoring the effect of sleep would simplify the data processing pipeline, facilitating the use of wearable sensors in both research and clinical practice.

**Supplementary Information:**

The online version contains supplementary material available at 10.1186/s12984-024-01384-z.

## Introduction

Wearable sensors have enormous potential to improve the delivery and outcomes of rehabilitation care [1, 2]. This potential is being realized in both the research and clinical care realms. Research labs are exploiting wearable sensor technology to measure activity outside the clinic/laboratory throughout a course of rehabilitation care [3, 4] or an experimental intervention [5–8]. These investigations have shown that improvements observed in the clinic/laboratory (i.e., activity *capacity*) do not necessarily translate to improvements in what a person actually does in their free-living environment (i.e., activity *performance*). While the use of these technologies has been primarily confined to research labs, their findings have resulted in a more recent emphasis to integrate these devices into clinical care settings [2, 9–11].

Despite the growing uptake of wearable sensors in rehabilitation, the use of these technologies pose significant burden on data collectors (i.e., patients, research participants) and analysts [1, 2, 11, 12]. For example, in the research setting, support personnel are needed to carefully inspect and process the data, requiring significant time and financial resources. The challenges become particularly problematic, if not unreasonable, when attempting to integrate these technologies into clinical settings. Busy clinicians do not have time to carefully scrutinize or perform manual computations of wearable sensor data. Thus, efforts to integrate these technologies into clinical care setting necessitate a fast, efficient, and least burdensome data processing pipeline.

Expediting the data processing pipeline to minimize burden on both research and clinical staff requires a thorough understanding of factors that may influence it [11–13]. One of the factors that is common among all persons and involves periodic movements is sleep [14]. The unexplored impact of sleep on the data processing pipeline has important consequences for both the development of research protocols and the resources required to process wearable sensor data. For example, some research protocols require participants to remove the wearable sensor during periods of sleep if sleep is not a primary interest [15]. This, however, requires participants to remember to don the device after periods of sleep, placing additional burden on the participant and increasing opportunities for data to go missing. On the contrary, protocols that permit participants to wear the device during sleep may increase burden on research support staff to remove periods of sleep if movement during sleep is not of interest. Understanding the effect of sleep on the wearable sensor data processing pipeline would determine whether these additional burdens are necessary and could streamline the use of these technologies in both research and clinical care settings.

The purpose of this investigation was to determine the effect of sleep on sensor-derived variables from upper limb accelerometry in people with and without upper limb impairment. We focused on the upper limb for two main reasons. The first is that upper limb impairment is a common sequelae of many conditions, such as stroke [16, 17], multiple sclerosis [18, 19], breast cancer [20, 21] and upper limb orthopedic conditions [22–24]. The second is that a common application of wearable sensor technology in rehabilitation research is to measure upper limb movement [25–28]. We therefore had two objectives for this analysis: (1) quantify the effect of sleep on sensor-derived variables from upper limb accelerometry, and (2) determine if the effect of sleep differs between people with and without upper limb impairment and across a two-day wearing period. Based on prior work demonstrating no difference in upper limb accelerometry variables across a two-day wearing period [29, 30], we did not anticipate that the effect of sleep would differ between recording days. However, demonstrating no difference does not prove that there is no effect. We therefore felt it prudent to include the effect of day in the model to allow us to test for these differences, should they exist. We also note that, among the multitude of variables that can be computed from upper limb accelerometry, it is currently unknown which are most important for future research and clinical practice. A previous study by Barth et al. found that variability in five upper limb accelerometry variables generated five unique clusters of individuals that differed in their upper limb use in daily life [31]. This suggests that these five variables may be important for measuring upper limb movement in daily life in people with and without upper limb impairment and serve as a starting point towards simplifying the measurement of upper limb movement in daily life using wearable sensors. We therefore report on these five variables in the primary paper and include 20 other variables that may be relevant for other scientific questions, populations, and clinical investigations in the supplement. The results of this analysis could help to minimize the personnel and computational burden to utilize wearable sensor technology in rehabilitation research labs and inform future efforts that seek to integrate this technology into clinical care settings.

## Methods

### Participants

This study was a secondary analysis of data collected during the baseline time point of a two-site (Washington University in St. Louis, MO and Shirley Ryan Ability Lab, Chicago, IL) prospective, longitudinal cohort study (NIH R37HD068290). This study recruits individuals without upper limb impairment (e.g., healthy controls) and individuals with upper limb impairment, including people with stroke, multiple sclerosis, distal radius fracture, proximal humerus and/or clavicle fracture, shoulder pain, or breast cancer who were undergoing physical and/or occupational therapy to improve upper limb function. Eligibility criteria for the control cohort included: (1) ≥ 18 years of age (2) free of neurologic, musculoskeletal, or medical conditions that affect the upper limb or significantly affect physical activity in general. Eligibility for the upper limb impairment cohort included: (1) ≥ 18 years of age (2) upper limb impairment as judged by a referring physician or surgeon (3) referral to rehabilitation services to address upper limb impairment (4) therapist documented goal(s) to increase or restore upper limb function (5) no other concurrent neurologic, musculoskeletal, or medical conditions that affect the upper limb or physical activity in general (6) no other co-morbid conditions determined by physician or therapy documentation that indicate a minimal chance for improvement in function (e.g., end-stage cancer diagnosis) (7) not pregnant or planning to become pregnant (8) no cognitive or communication problems that would prevent them from completing the study. For this analysis, all participants with upper limb impairment were grouped together as we had no scientific reason to suspect that the effects of sleep on the upper limb performance variables would differ based on diagnosis, nor would we have the statistical power to detect these sub-group differences. Only participants who wore the sensors for the full 48-hour wear period were included in this analysis. The study was approved by Washington University’s Institutional Review Board, and all participants signed informed consent prior to engaging in any study procedures. This manuscript was developed in accordance with the STROBE guidelines.

### Measures

All study procedures occurred remotely and study data were collected and managed using REDCap electronic data capture tools housed at Washington University in St. Louis. REDCap (Research Electronic Data Capture) is a secure, web-based software platform designed to support data capture for research studies providing (1) an intuitive interface for validated data capture; (2) audit trails for tracking data manipulation and export procedures; (3) automated export procedures for seamless data downloads to common statistical packages; and (4) procedures for data integration and interoperability with external sources [32, 33]. Participants in both cohorts were sent several surveys and two wrist-worn accelerometers to wear for a 48-hour period. The surveys collected demographic information, including age, sex, race, and ethnicity, as well as clinical information (upper limb impairment cohort only). Surveys were completed online or on paper, depending on the participant’s preference, and within one week of wearing the sensors. For the upper limb impairment cohort, participants completed the baseline time point within two weeks of starting outpatient rehabilitation services.

Upper limb accelerometry variables were measured using established, reliable, and valid bilateral wrist-worn accelerometry methodology [34, 35]. Participants wore tri-axial GT9X Link accelerometers (ActiGraph Inc, Pensacola, Florida) on each wrist for 48 h that sampled at 30 Hz. Participants were instructed to go about their normal routine while wearing the sensors and to keep the sensors on during sleep. Once the sensors were returned to the lab, the data were visually inspected to verify a 48-hour wear period using ActiLife 6 software (ActiGraph Inc, Pensacola, Florida) and exported for further processing.

### Upper Limb performance variables

Work is ongoing to determine which sensor-derived variables from upper limb accelerometry are most important for research and clinical practice [31]. Thus, we examined 25 variables in total that reflect different aspects of motor behavior of the upper limbs in daily life under the assumption that the relevance of specific variables may vary across research questions, clinical populations, and scientific fields [2, 29, 36–38]. Here, we focus on five variables that were previously shown to generate unique clusters of individuals with and without neurologic upper limb deficits [31], and present the remaining 20 variables in the Supplement. Table 1 displays the five upper limb sensor variables and their respective calculations.

**Table 1:** Description of Upper Limb Sensor Variables*

Variable	Description
Preferred Time	Time (in hours) that the dominant/unaffected limb is moving.
Non-Preferred Time	Time (in hours) that the non-dominant/affected limb is moving.
Use Ratio	Ratio of hours of non-dominant/affected limb movement, relative to hours of dominant/unaffected limb movement.
Non-Preferred Magnitude	Median of the accelerations of the non-dominant/affected limb, in activity counts
Non-Preferred Magnitude Standard Deviation	Standard deviation of the magnitude of accelerations across the non-dominant/affected limb, in activity counts

*Preferred indicates the dominant limb (control cohort) or unaffected limb (upper limb impairment cohort). *Non-preferred* indicates the non-dominant limb (control cohort) or affected limb (upper limb impairment cohort). If a participant identified both limbs as affected, their clinical documentation was reviewed to determine which limb was more affected. The more affected limb was considered the non-preferred limb in all variable calculations

Two data files were extracted from ActiLife 6 software (ActiGraph Inc, Pensacola, Florida): a raw 30 Hz acceleration file (in gravitational units) and down-sampled 1 Hz data file (in Actigraph activity counts). The 30 Hz data were band-pass filtered from 0.2 to 12 Hz to remove acceleration components incompatible with human activity. Data in the 1 Hz file were first filtered using ActiGraph’s proprietary filtering algorithm, which uses a maximum gain of 0.759 Hz and goes down to -6dB at 0.212 Hz at 2.148 Hz and then down-sampled from 30 Hz to 1-second epochs for each axis by summing the 30 samples within each second [39]. Accelerations in each axis were combined into a single vector magnitude using the formula $\sqrt{{X}^{2}+{Y}^{2}+{Z}^{2}}$. A vector magnitude threshold of ≥ 2 activity counts was used to determine if the upper limb was active for each 1-second epoch [40, 41]. To compute the variables preferred time and non-preferred time, seconds of movement that exceeded this threshold were summed over the wearing period and converted to hours. The use ratio was calculated as the ratio of non-preferred limb movement relative to the preferred limb movement. The non-preferred magnitude and standard deviation (Table 1) are reported in activity counts [39].

### Sleep detection

Actigraphy is a reliable and valid method for detecting sleep, with previous reports demonstrating acceptable to high agreement with polysomnography [42–45]. For this study, we employed a multi-step approach to detect sleep using published algorithms [42, 43]. Sleep time was determined from the sensor worn on the participant’s preferred upper limb using the methodology described by Schoch and colleagues [43]. Briefly, this approach applies the Sadeh sleep algorithm [42] and includes several additional adjustments to improve the algorithm’s accuracy [43]. The first adjustment applies a criterion to distinguish between sleep and wake time using the mean vector magnitude for each day of data multiplied by a threshold. Here, we used a threshold of 0.35 because it was best suited to identify sleep in pilot testing. The second adjustment removes periods when the sensors were not worn, which was not applicable for this analysis as all participants wore the sensors for the full 48-hour wear period. The third adjustment utilized criteria by Webster et al. [46] to address instances of incorrectly identifying periods of sleep while the participant was awake by applying five smoothing routines. For example, brief periods of less than 10 min of sleep surrounded by at least 20 min of wake before and/or after were rescored as wake. The final adjustment involved generating a sleep graph from the previous adjustments and displaying it to the research team member processing the data. The research team member made additional adjustments (if needed) based on any notes made by the participant on their accelerometry wearing log.

For each sensor variable of interest and wearing day, the sensor variable was computed using the full wear time (sleep included) and with sleep time removed (sleep excluded). The effect of sleep was quantified as the difference between sleep included and excluded (i.e., sleep included – sleep excluded). Custom-written R scripts (R Core Team 2021, version 4.2.1) were used for all variable computations and to implement the sleep detection algorithms described above [47, 48].

### Statistical analysis

Linear mixed effects regression (LMER) was used to address our two study objectives and account for within-participant repeated accelerometry measurement across days [49]. For each sensor variable, a LMER model was tested in which the difference between sleep included and excluded was the dependent variable. Each model included a random intercept for participant and fixed effects for day and cohort and their interaction:

Difference_(Included – Excluded)ij_ = β_0_ + γ_0i_ + β_1_Cohort + β_2_Day_j_ + β_3_(Cohort*Day_j_) + Ɛ_ij_.

Our first objective, to quantify the effect of sleep on each sensor variable, was addressed by interpreting the β_0_ parameter of the model. Using the difference between sleep included and excluded as the outcome allowed us to interpret the intercept (β_0_) as the main-effect of including sleep in the algorithm. A statistically significant p -value for the intercept thus shows that including/excluding has a non-zero effect on the output.

Our second objective, to determine if the effect of sleep varied by cohort (people with upper limb impairment vs. without) and by day, was addressed by interpreting the effects of cohort (β_1_Cohort), day (β_2_Day), and their interaction (β_3_(Cohort*Day)). All fixed effects were contrast coded so that regression slopes can be interpreted as main effects (e.g., β_1_ is the effect of Cohort on average across days). Confidence intervals were computed using bootstrapping, and *P* values were adjusted using the Benjamini-Hochberg false discovery rate (FDR) [50] procedure to account for the number of statistical tests performed on all 25 variables (25 variables x 4 effects per LMER model). All LMER models were conducted in R (R Core Team 2021, version 4.2.1) using the lmer [51], AICcmodavg [52], and lmerTest [53] packages.

## Results

At the time of this analysis, 148 individuals met the eligibility criteria to participate in the larger study. Of these 148, 16 individuals had missing data due to withdrawal from the study or loss to follow-up, and 5 individuals did not meet the 48-hour wear criteria for this analysis. This resulted in 127 individuals (65 without upper limb impairment and 62 with upper limb impairment) included in this analysis. Table 2 displays the demographic and clinical characteristics of our full sample as well as each cohort. Supplemental Table 1 displays the demographic and clinical characteristics of each upper limb condition group.

**Table 2:** Characteristics of Study Sample

Variable	Full Sample (*n* = 127)	Controls (*n* = 65)	UL Conditions (*n* = 62)
Age (yrs)	53.5 ± 17.8	49.4 ± 20.1	57.8 ± 13.9
Sex			
Female	62.2% (79)	69.23% (45)	54.84% (34)
Male	37.80% (48)	30.77% (20)	45.16% (28)
Race			
White	71.65% (91)	80.0% (52)	62.9% (39)
Black	21.26% (27)	10.77% (7)	32.26% (20)
Asian	5.51% (7)	9.23% (6)	1.61% (1)
Native Hawaiian or Pacific Islander	0.79% (1)	0% (0)	1.61% (1)
Ethnicity			
Hispanic, Latino	3.15% (4)	1.54% (1)	4.84% (3)
Non-Hispanic, Non-Latino	96.85% (123)	98.46% (64)	95.16% (59)
Hand Dominance			
Right	90.55% (115)	90.77% (59)	90.32% (56)
Left	8.66% (11)	9.23% (6)	8.06% (5)
Both	0.79% (1)	0% (0)	1.61% (1)
Employment			
Working ≥ 37.5 h/wk	37.01% (47)	49.23% (32)	24.19% (15)
Working ≥ 20 h/wk	5.51% (7)	6.15% (4)	4.84% (3)
Working < 20 h/wk	7.87% (10)	10.77% (7)	4.84% (3)
Not working	49.61% (63)	33.85% (22)	66.13% (41)
Living Situation			
Living alone, assistance with BADLs	1.57% (2)	0% (0)	3.23% (2)
Living alone, independent with BADLs	24.41% (31)	27.69% (18)	20.97% (13)
Living with others, assistance with BADLs	7.09% (9)	0% (0)	14.52% (9)
Living with others, independent with BADLs	66.93% (85)	72.31% (47)	61.29% (38)
Affected Side			
Right			48.39% (30)
Left			45.16% (28)
Both			6.45% (4)
Concordance*			
Yes			45.16% (28)
No			54.84% (34)

Abbreviations: UL- upper limb, BADLs- basic activities of daily living. *Concordance is when the dominant upper limb is the affected limb

**Fig. 1:**
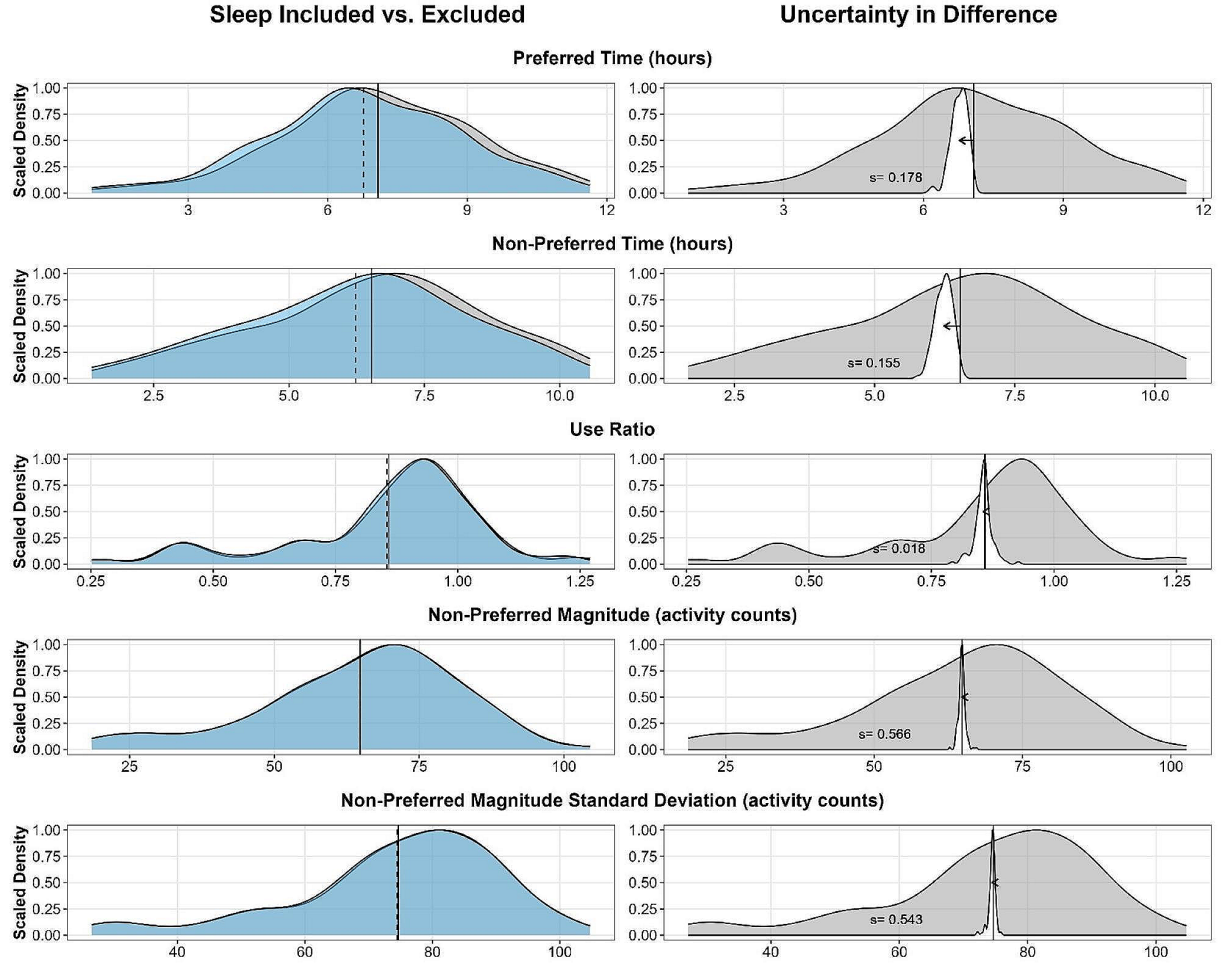
Differences between sleep included and excluded for each sensor variable. *Left panel*: Overlapping density plots displaying the distribution of each sensor variable with sleep included (grey) and sleep excluded (blue). The mean for each distribution is shown as a thin vertical line (solid- sleep included, dashed- sleep excluded). *Right panel*: Overlapping density plots displaying the distribution of each sensor variable with sleep included (grey) and the differences between sleep included and excluded (white) scaled and centered around the variable mean with sleep included. The thin vertical line displays the mean of the distribution with sleep included. The arrow depicts the direction the variable would shift by excluding sleep. The number (s = ) in each plot represents the standard deviation of the differences between sleep included and excluded for that variable, demonstrating uncertainty in the difference. Preferred indicates the dominant upper limb (Controls) or the unaffected upper limb (Upper Limb Impairment cohort)

Our first objective examined the effect of sleep on each sensor variable. We found a statistically significant difference between sleep included and excluded for three of the five sensor variables: preferred time, non-preferred time, and non-preferred magnitude standard deviation (Table 3, intercept values). To help contextualize these differences, the left column of Fig. 1 displays overlapping density plots of sleep included (grey) and sleep excluded (blue) for each of the five sensor variables. The right column of Fig. 1 displays the density plot for sleep included (grey), which is then overlain by the distribution of the differences between sleep included and excluded (white density plot, vertically scaled and centered around the mean of the variable with sleep included). For example, looking at preferred time (Fig. 1, top left), one can see that the distribution for sleep excluded (blue) is slightly shifted to the left compared to sleep included (grey), suggesting that excluding sleep results in a reduction in preferred time hours compared to including sleep. Correspondingly, the top right panel of Fig. 1 shows the distribution of differences is shifted down by 0.31 h relative to the mean when sleep is included (solid line vs. direction and length of arrow), but there is uncertainty in that effect, with the standard deviation of the differences being 0.18. Thus, for any given point in the black histogram, we would expect that point to be shifted by 0.31 ± 0.18 h (or 19 ± 11 min) if sleep was to be included.

**Table 3:** Linear Mixed Effects Regression Model Parameters for each Sensor Variable

Sensor Variable	Model Parameter	Estimate	95% Confidence Interval	T-Value	FDR Adj. *P*-Value
*Preferred Time*	Intercept	0.31	0.28–0.35	19.81	*< 0.001*
	Day	-0.02	-0.07–0.02	-1.04	0.61
	Cohort	0.01	-0.05–0.07	0.18	0.94
	Day x Cohort	0.02	-0.07–0.11	0.49	0.78
*Non-Preferred Time*	Intercept	0.29	0.26–0.32	21.42	*< 0.001*
	Day	-0.03	-0.07–0.01	-1.49	0.44
	Cohort	-0.03	-0.09–0.02	-1.24	0.58
	Day x Cohort	0.01	-0.08–0.11	0.22	0.92
*Use Ratio*	Intercept	0.004	0.00007–0.01	2.23	0.13
	Day	-0.001	-0.01–0.004	-0.39	0.82
	Cohort	0.0004	-0.01–0.01	0.12	0.94
	Day x Cohort	0.01	-0.001–0.02	1.59	0.40
*Non-Preferred Magnitude*	Intercept	0.04	-0.06–0.14	0.82	0.71
	Day	0.04	-0.14–0.20	0.49	0.78
	Cohort	-0.3	-0.49 – -0.13	-3.13	*0.02*
	Day x Cohort	0.01	-0.33–0.32	0.10	0.94
*Non-Preferred Magnitude SD*	Intercept	0.2	0.1–0.3	4.17	*0.001*
	Day	0.09	-0.01–0.2	1.65	0.38
	Cohort	-0.06	-0.3–0.13	-0.64	0.74
	Day x Cohort	-0.09	-0.32–0.14	-0.76	0.72

**Fig. 2:**
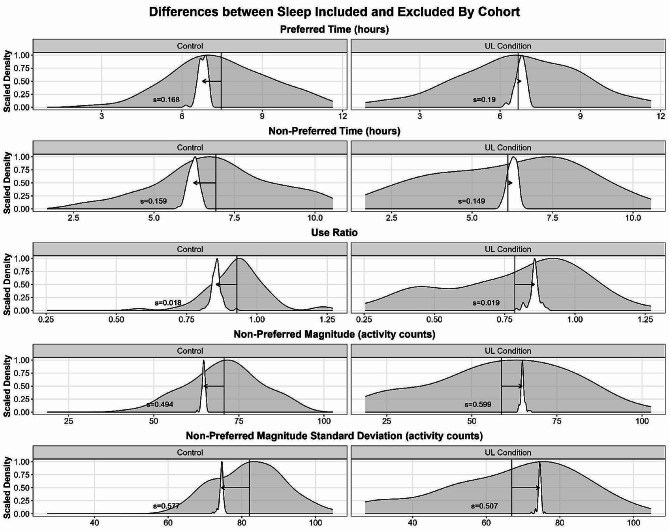
Differences between sleep included and excluded for each sensor variable by cohort. Overlapping density plots displaying the distribution of each sensor variable with sleep included (grey) and the differences between sleep included and excluded (white, scaled and centered around the variable mean with sleep included) for Control participants without UL impairment (left) and people with UL impairment (right). The thin vertical line displays the mean of the distribution with sleep included. The arrow depicts the direction the variable would shift by excluding sleep. The number (s = ) in each plot represents the standard deviation of the differences between sleep included and excluded for that variable, demonstrating uncertainty in the difference. Preferred indicates the dominant upper limb (Controls) or the unaffected upper limb (Upper Limb Impairment cohort). *Abbreviations: UL- upper limb*

**Fig. 3:**
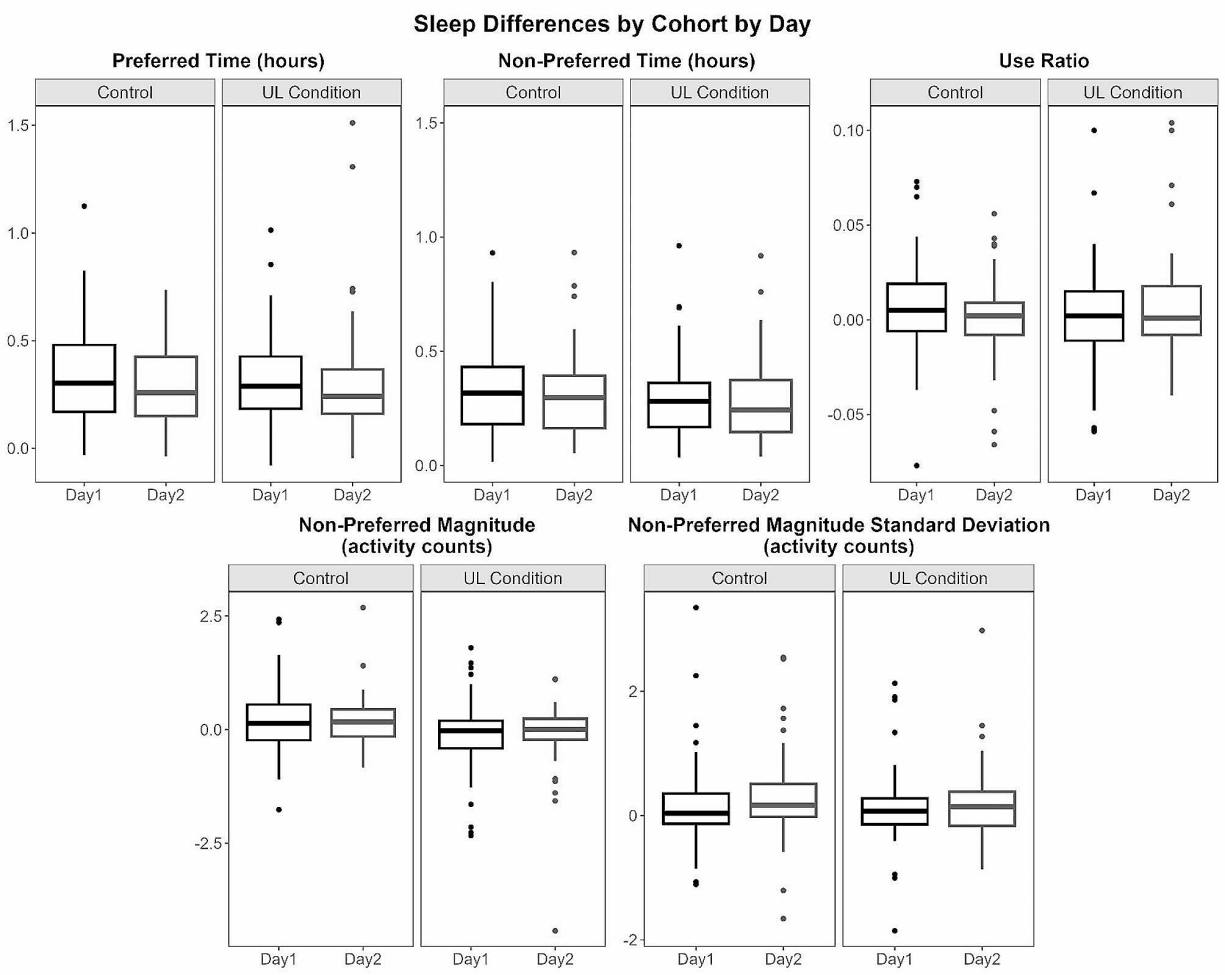
Differences between sleep included and excluded for each sensor variable by cohort and by day. Each coupled panel represents a sensor variable, with the leftward panel representing Control participants and the rightward panel representing participants with UL impairment. Each panel displays box plots of the differences between sleep included and excluded for Day 1 and Day 2. Preferred indicates the dominant upper limb (Controls) or the unaffected upper limb (Upper Limb Impairment cohort)

Our second objective examined if the effect of sleep differed between individuals with and without upper limb impairment and between wearing days. Non-preferred magnitude was the only variable in which we observed a significant difference between people with and without upper limb impairment (Table 3). Compared to people without upper limb impairment (i.e., controls), people with upper limb impairment had a slightly smaller difference between sleep included and excluded for this variable. Despite being statistically significant, this difference between cohorts appears to be trivially small, see Fig. 2. Figure 2 displays overlapping density plots of sleep included (grey) and the distribution of differences between sleep included and excluded (white density plot, scaled and centered around the variable mean with sleep included for interpretability) for each cohort. Overall, the effect of sleep for people with and without upper limb impairment was relatively similar, as evidenced by the Cohort estimates in Table 3 and in examining the distributions of Fig. 2. The effect of day was not significant for any of the LMER models (Table 3), suggesting that the effect of sleep was not substantially different across wearing days. There was also no significant interaction between day and cohort for any of the upper limb performance variables, suggesting that the effects of sleep were relatively consistent between cohorts and wearing days (Fig. 3).

Supplemental Tables 2–21 and Supplemental Figs. 1–20 display the LMER model results and visual representations of the findings for each supplemental variable, respectively.

## Discussion

The purpose of this study was to investigate the effect of sleep on the wearable sensor data processing pipeline to facilitate the use of wearable sensor technology in rehabilitation research and clinical care settings. Our first objective was to quantify the effect of sleep on sensor-derived variables from upper limb accelerometry. We observed a small, significant difference between sleep included and excluded for three of the five sensor variables: preferred time, non-preferred time, and non-preferred magnitude standard deviation. Our second objective was to determine if the effect of sleep differed between people with and without upper limb impairment and across a two-day wearing period. Here, we found that the effect of sleep differed slightly between people with and without upper limb impairment for one of the five variables, non-preferred magnitude, and that the effect of sleep was not different across a two-day wearing period. These findings have important implications for the use of wearable sensors in both research and clinical care settings.

Despite being statistically significant, the differences we observed were small. For example, excluding sleep reduces non-preferred time by 0.29 ± 0.15 (or 17.4 ± 9 min) relative to the mean when sleep is included. A difference of 17 min of non-preferred upper limb use is likely small in many contexts. The mean number of minutes of non-preferred time (with sleep included) of the sample was 392. To put this difference into context, 17 min of non-preferred time equates to 4.3% of the mean non-preferred time of the sample. The differences in non-preferred magnitude and its standard deviation were even smaller (Table 3; Figs. 1 and 2). Collectively, these data suggest that the effect of sleep on sensor-derived variables from upper limb accelerometry is small and similar between people and without upper limb impairment and across a two-day wearing period and can likely be ignored in most cases.

From a research perspective, these findings may help minimize burden on both research staff and participants [2]. For research staff, committing the time and resources to remove periods of sleep when processing accelerometry data from the upper limb(s) is likely not necessary in most cases. For research participants, removing a sensor opens the possibility that a participant will forget to put the sensor back on, creating opportunities for data to go missing [54]. These results suggest that research protocols need not instruct participants to remove the sensors during sleep, which may improve participant adherence to wearing the devices [15]. Some research protocols also instruct participants to record their activities while wearing the sensor(s) [55]. If sleep is not a behavior of interest in a research study using accelerometry to measure upper limb movement, there is likely no need to burden participants with recording sleep activity. This is not to suggest that activity diaries as a whole are not necessary, as diaries can be helpful for comparing wearable sensor data to the participant’s reported activity. These reduced burdens will streamline the use of wearable sensor technology in research labs already accustomed to it, and also bring this technology within reach of labs unfamiliar to it, through a less computationally complex data processing pipeline.

From the clinical perspective, the success of deploying new technologies in clinical care settings hinges upon how well the technology is integrated into clinical workflows [56–60]. This includes making the data directly available in the electronic health record (EHR) [1, 56, 61, 62]. Busy clinicians do not have time to process raw data or generate summary variables. It is therefore imperative that the data processing pipeline be as simple and efficient as possible. Findings from this study suggest that the data processing pipeline can be simplified by ignoring periods of sleep when deploying wearable sensors to compute variables that reflect motor behavior of the upper limbs in people with and without upper limb impairment. This will expedite data processing, minimize burden on the clinician, and allow summary data to be in the hands of busy clinicians sooner. We also note that, in scenarios in which data are collected over multiple time points, the decision to remove periods of sleep or not should remain consistent, regardless of whether the data processing pipeline is built for research or clinical care initiatives.

There are several limitations to consider when interpreting the results of this study. The first is that wrist-worn actigraphy is not without measurement error [54, 63]. Using wrist-worn actigraphy to detect sleep relies on the use of upper limb movements to determine whether periods of sleep have occurred [64]. Any missed or incorrectly identified upper limb movements would have a downstream effect on a sleep algorithm’s ability to quantify sleep patterns. Actigraphy-based sleep detection algorithms themselves also suffer from accuracy challenges [65, 66]. For example, if a person is awake but not moving their upper limbs, the algorithm is likely to incorrectly identify this period as sleep. For these reasons, actigraphy-based sleep detection algorithms tend to perform better at detecting sleep (i.e., higher sensitivity), rather than periods of wakefulness (i.e., lower specificity) [44, 66, 67]. This means we may have over-estimated the amount of sleep in this study and potentially over-estimated the effect of sleep on the upper limb sensor variables. For this study, the Sadeh sleep algorithm was selected due to its high agreement with polysomnography and widespread use in the literature [42, 66]. While other algorithms exist, they tend to perform similarly in terms of overall accuracy, sensitivity, and specificity [66]. It is therefore plausible that using a different algorithm would have yielded a similar result; however, this was not formally tested in this study.

## Conclusions

The effect of sleep on sensor-derived variables from upper limb accelerometry is small and similar between people with and without upper limb impairment and across a two-day wearing period. This small effect can likely be ignored in the upper limb wearable sensor data processing pipeline in many situations. To continue to support the uptake of wearable sensor technology in rehabilitation research and clinical care, future work should investigate the role of other factors, such as upper limb movement during walking, on the data processing pipeline. Efforts should also be directed towards understanding which sensor-derived variables from upper limb accelerometry are most important and should be carried forward in future research and clinical care initiatives.

### Electronic supplementary material

Below is the link to the electronic supplementary material.

Supplementary Material 1

## Data Availability

The datasets analyzed during the current study are available from the corresponding author upon request.

## Abbreviations

REDCap: Research Electronic Data Capture
LMER: Linear mixed effects regression
UL: upper limb
BADLs: basic activities of daily living
EHR: electronic health record
