# Supplementary material for: Quantifying the effects of sleep on sensor-derived variables from upper limb accelerometry in people with and without upper limb impairment
Source: J Neuroeng Rehabil. 2024 May 28;21:86. doi: 10.1186/s12984-024-01384-z (PMC11131201; doi:10.1186/s12984-024-01384-z)
Supplement: Supplementary file 1 — Supplementary Material 1 [file 12984_2024_1384_MOESM1_ESM.docx]

Quantifying the Effects of Sleep of Sensor-Derived Variables from Upper Limb Accelerometry in People with and without Upper Limb Impairment

SUPPLEMENTAL MATERIAL

Supplemental Table 1. Demographic and Clinical Characteristics by Diagnosis

| **Variable** | **Controls**  **(n = 65)** | **Stroke**  **(n = 25)** | **Multiple Sclerosis**  **(n = 9)** | **Shoulder Pain**  **(n = 10)** | **Fracture, Distal Radius**  **(n = 9)** | **Fracture, Proximal Humerus**  **(n = 5)** | **Breast Cancer**  **(n = 4)** |
| --- | --- | --- | --- | --- | --- | --- | --- |
| Age (yrs) | 49.42 ± 20.07 | 62.28 ± 15.12 | 46.33 ± 9.82 | 54.40 ± 13.22 | 58.56 ± 11.78 | 60.00 ± 14.02 | 59.75 ± 9.57 |
| Sex  Female  Male | 69.2% (45)  30.8% (20) | 36% (9)  64% (16) | 77.8% (7)  22.2% (2) | 60% (6)  40% (4) | 77.8% (7)  22.2% (2) | 20% (1)  80% (4) | 100% (4)  0% (0) |
| Race  White  Black  Asian  Native Hawaiian or Pacific Islander | 80% (52)  10.8% (7)  9.2% (6)  0% (0) | 44% (11)  56% (13)  0% (0)  0% (0) | 44.4% (4)  44.4% (4)  0% (0)  11.1% (1) | 100% (10)  0% (0)  0% (0)  0% (0) | 88.9% (8)  11.1% (1)  0% (0)  0% (0) | 80% (4)  0% (0)  20% (1)  0% (0) | 50% (2)  50% (2)  0% (0)  0% (0) |
| Ethnicity  Hispanic, Latino  Non-Hispanic, Non-Latino | 1.5% (1)  98.5% (64) | 8% (2)  92% (23) | 11.1% (1)  88.9% (8) | 0% (0)  100% (10) | 0% (0)  100% (9) | 0% (0)  100% (5) | 0% (0)  100% (4) |
| Hand Dominance  Right  Left  Both | 90.8% (59)  9.2% (6)  0% (0) | 88% (22)  8% (2)  4% (1) | 100% (9)  0% (0)  0% (0) | 90% (9)  10% (1)  0% (0) | 88.9% (8)  11.1% (1)  0% (0) | 100% (5)  0% (0)  0% (0) | 75% (3)  25% (1)  0% (0) |
| Employment  Working ≥ 37.5 hrs/wk  Working ≥ 20 hrs/wk  Working < 20 hrs/wk  Not working | 49.2% (32)  6.2% (4)  10.8% (7)  33.8% (22) | 8% (2)  0% (0)  0% (0)  92% (23) | 11.1% (1)  0% (0)  0% (0)  88.9% (8) | 70% (7)  0% (0)  10% (1)  20% (2) | 11.1% (1)  11.1% (1)  22.2% (2)  55.6% (5) | 60% (3)  40% (2)  0% (0)  0% (0) | 25% (1)  0% (0)  0% (0)  75% (3) |
| Living Situation  Living alone, assistance with BADLs  Living alone, independent with BADLs  Living with others, assistance with BADLs  Living with others, independent with BADLs | 0% (0)  27.7% (18)  0% (0)  72.3% (47) | 0% (0)  16% (4)  32% (8)  52% (13) | 22.2% (2)  22.2% (2)  0% (0)  55.6% (5) | 0% (0)  10% (1)  0% (0)  90% (9) | 0% (0)  44.4% (4)  0% (0)  55.6% (5) | 0% (0)  20% (1)  20% (1)  60% (3) | 0% (0)  25% (1)  0% (0)  75% (3) |
| Affected Side  Right  Left  Both |  | 48% (12)  48% (12)  4% (1) | 44.4% (4)  44.4% (4)  11.2% (1) | 60% (6)  40% (4)  0% (0) | 33.3% (3)  66.7% (6)  0% (0) | 80% (4)  20% (1)  0% (0) | 25% (1)  25% (1)  50% (2) |
| Concordance*  Yes  No |  | 40% (10)  60% (15) | 55.6% (5)  44.4% (4) | 50% (5)  50% (5) | 44.4% (4)  55.6% (5) | 80% (4)  20% (1) | 50% (2)  50% (2) |

For each of the figures below: **A)** *Left panel-* Overlapping density plots displaying the distribution of the sensor variable with sleep included (grey) and sleep excluded (blue). The mean for each distribution is shown as a thin vertical line (solid- sleep included, dashed- sleep excluded). *Right panel-* Overlapping density plots of sleep included (grey) and the differences between sleep included and excluded (white), scaled and centered around the variable mean with sleep included. The thin vertical line displays the mean of the distribution with sleep included. The arrow depicts the direction the variable would shift by excluding sleep. The number (s = ) in each plot represents the standard deviation of the differences between sleep included and excluded for that variable, demonstrating uncertainty in the difference. **B)** Overlapping density plots of sleep included (grey) and the differences between sleep included and excluded (white, scaled and centered around the variable mean with sleep included) for Control participants without upper limb impairment *(left panel)* and people with upper limb impairment *(right panel)*. **C)** Box plots of the differences between sleep included and excluded for Day 1 and Day 2 for Controls *(left panel)* and participants with upper limb impairment *(right panel)*. For all figures, Preferred indicates the dominant upper limb (Controls) or the unaffected upper limb (Upper Limb Impairment cohort). **Concordance is when the dominant upper limb is the affected upper limb.*


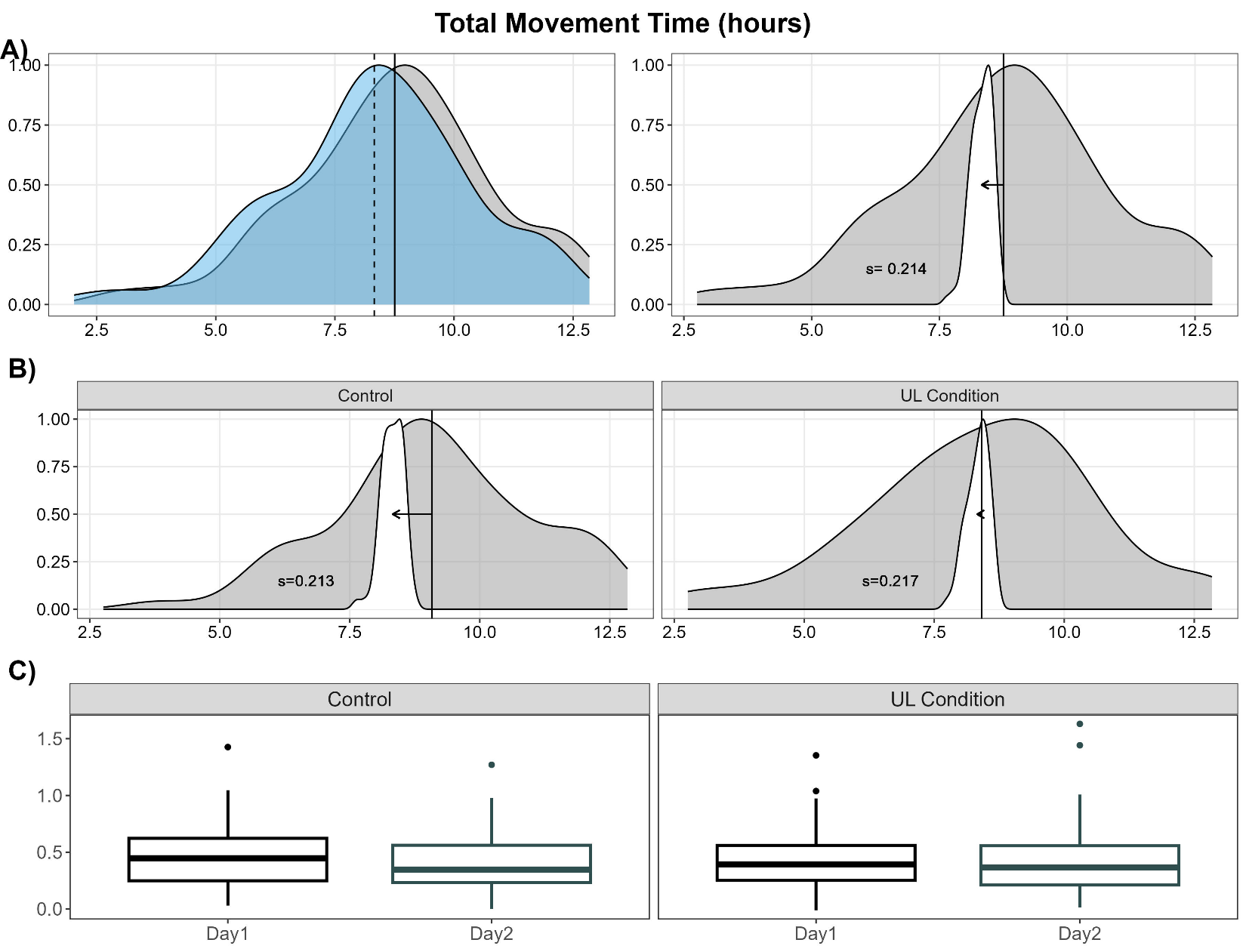


**Supplemental Figure 1. Effect of Sleep on Total Movement Time.** Total movement time is the time (in hours) that either the preferred limb, non-preferred limb, or both are moving.

| **Sensor Variable** | **Model Parameter** | **Estimate** | **95% Confidence Interval** | **T-Value** | **FDR Adj. P-Value** |
| --- | --- | --- | --- | --- | --- |
| Total Movement Time | Intercept | 0.43 | 0.39 – 0.47 | 22.61 | <0.001 |
|  | Day | -0.03 | -0.10 – 0.02 | -1.13 | 0.61 |
|  | Cohort | -0.02 | -0.09 – 0.05 | -0.47 | 0.79 |
|  | Day x Cohort | 0.05 | -0.09 – 0.18 | 0.76 | 0.72 |

Supplemental Table 2. Linear Mixed Effects Regression Results for Total Movement Time


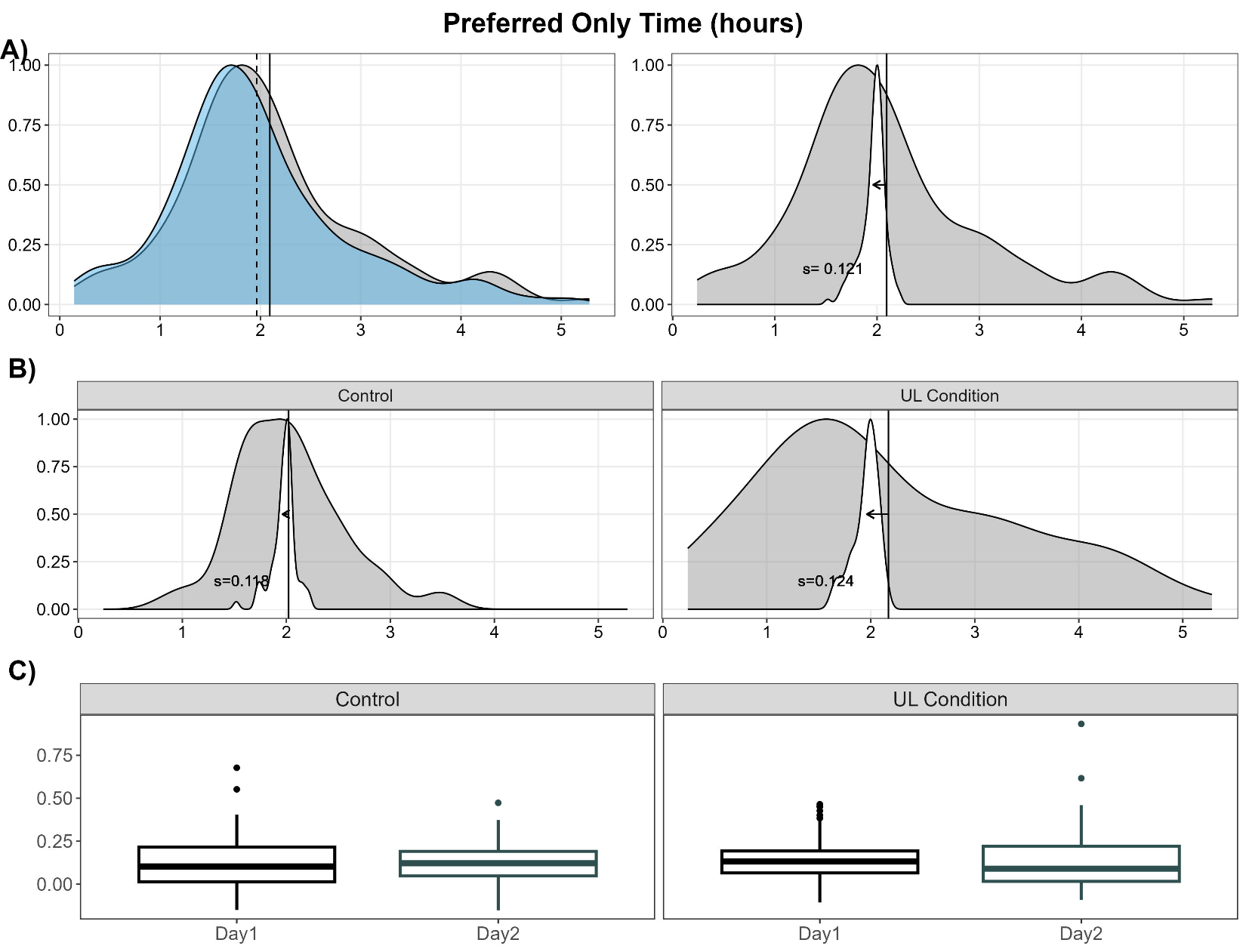


**Supplemental Figure 2. Effect of Sleep on Preferred Only Time.** Preferred only time is the time (in hours) that the preferred limb is moving, while the non-preferred limb is still.

Supplemental Table 3. Linear Mixed Effects Regression Results for Preferred Only Time

| **Sensor Variable** | **Model Parameter** | **Estimate** | **95% Confidence Interval** | **T-Value** | **FDR Adj. P-Value** |
| --- | --- | --- | --- | --- | --- |
| Preferred Only Time | Intercept | 0.13 | 0.11 – 0.15 | 12.29 | <0.001 |
|  | Day | -0.002 | -0.03 – 0.03 | -0.13 | 0.94 |
|  | Cohort | 0.02 | -0.02 – 0.07 | 0.99 | 0.61 |
|  | Day x Cohort | -0.02 | -0.08 – 0.05 | -0.53 | 0.78 |


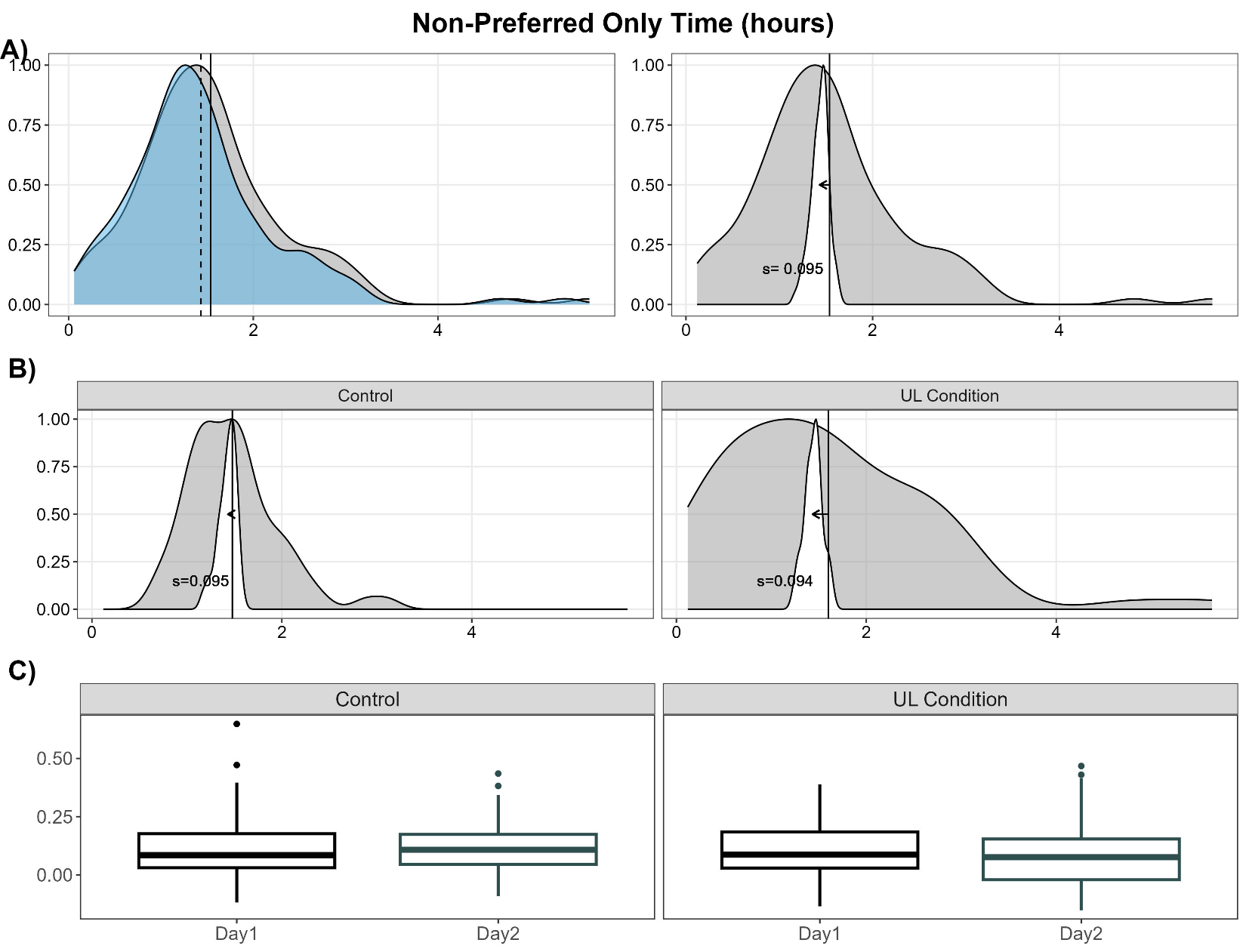


**Supplemental Figure 3. Effect of Sleep on Non-Preferred Only Time.** Non-preferred only time is the time (in hours) that the non-preferred limb is moving, while the preferred limb is still.

Supplemental Table 4. Linear Mixed Effects Regression Results for Non-Preferred Only Time

| **Sensor Variable** | **Model Parameter** | **Estimate** | **95% Confidence Interval** | **T-Value** | **FDR Adj. P-Value** |
| --- | --- | --- | --- | --- | --- |
| Non-Preferred Only Time | Intercept | 0.10 | 0.09 – 0.12 | 12.56 | <0.001 |
|  | Day | -0.006 | -0.03 – 0.02 | -0.45 | 0.79 |
|  | Cohort | -0.02 | -0.05 – 0.01 | -1.19 | 0.61 |
|  | Day x Cohort | -0.02 | -0.08 – 0.04 | -0.74 | 0.72 |


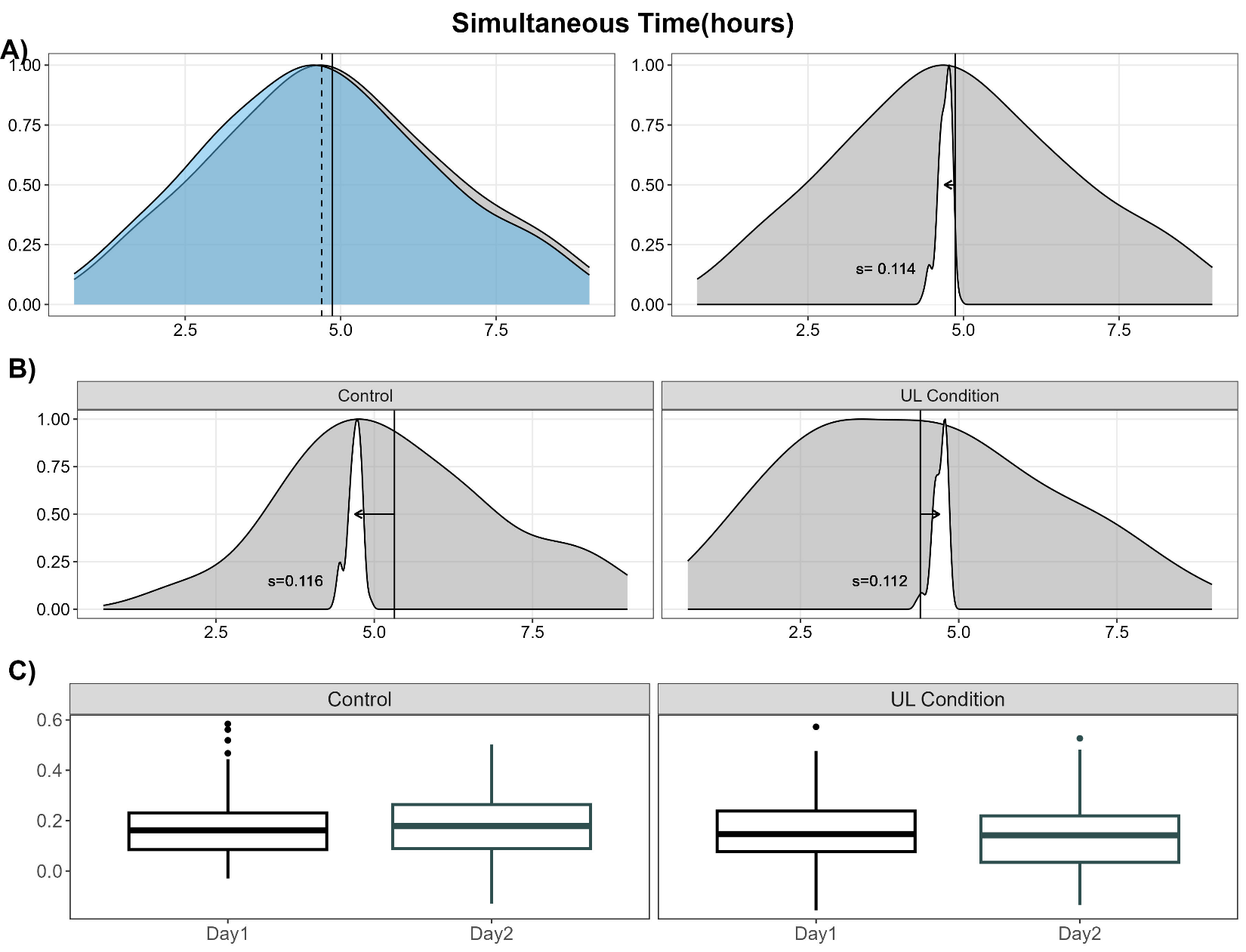


**Supplemental Figure 4. Effect of Sleep on Simultaneous Time.** Simultaneous time is the time (in hours) that both limbs are moving.

Supplemental Table 5. Linear Mixed Effects Regression Results for Simultaneous Time

| **Sensor Variable** | **Model Parameter** | **Estimate** | **95% Confidence Interval** | **T-Value** | **FDR Adj. P-Value** |
| --- | --- | --- | --- | --- | --- |
| Simultaneous Time | Intercept | 0.17 | 0.15 – 0.19 | 16.96 | <0.001 |
|  | Day | -0.01 | -0.03 – 0.02 | -0.62 | 0.74 |
|  | Cohort | -0.02 | -0.05 – 0.02 | -1.03 | 0.61 |
|  | Day x Cohort | -0.02 | -0.07 – 0.03 | -0.79 | 0.72 |


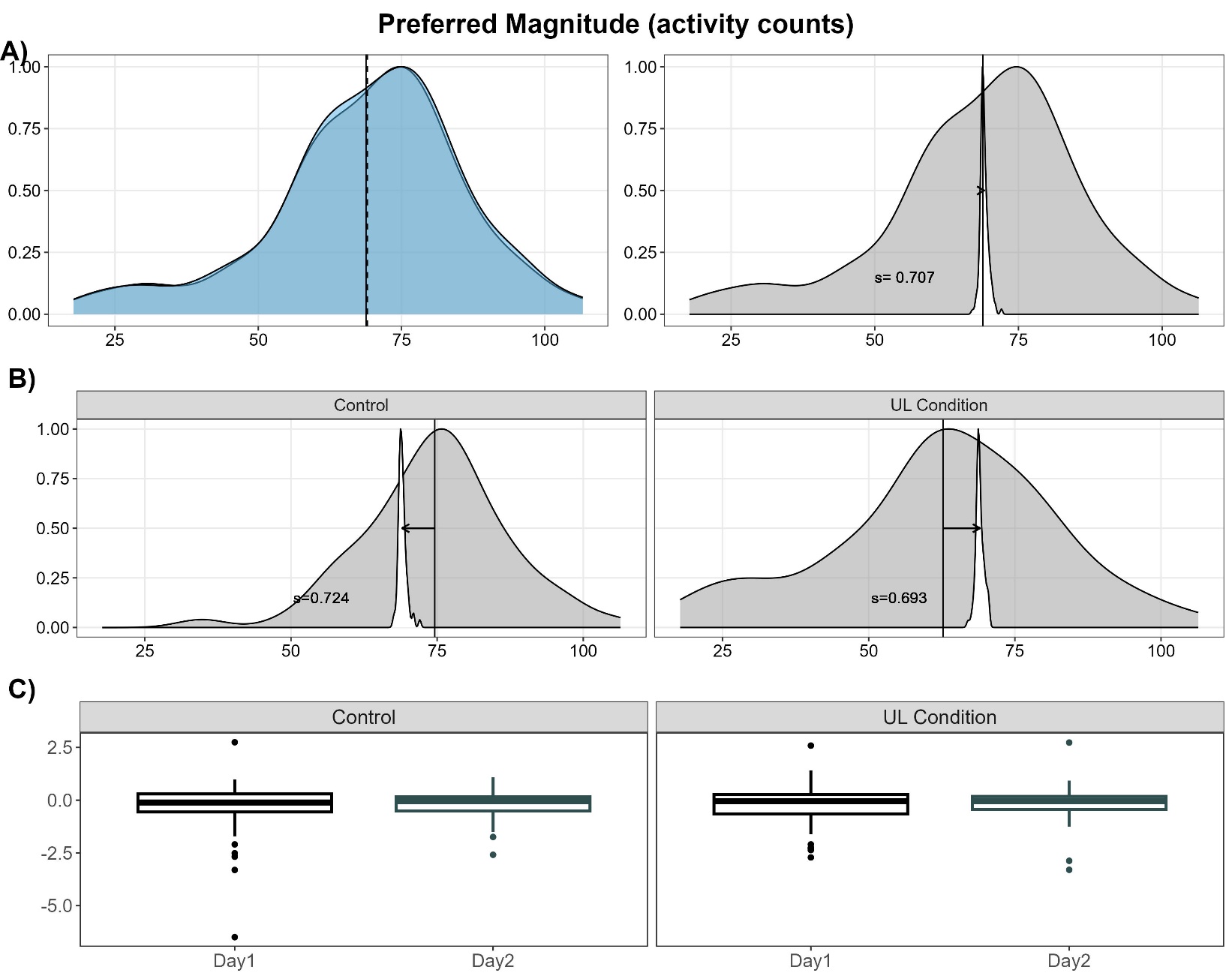


**Supplemental Figure 5. Effect of Sleep on Preferred Magnitude.** Preferred magnitude is the intensity, or magnitude of accelerations of the preferred limb, in activity counts.

Supplemental Table 6. Linear Mixed Effects Regression Results for Preferred Magnitude

| **Sensor Variable** | **Model Parameter** | **Estimate** | **95% Confidence Interval** | **T-Value** | **FDR Adj. P-Value** |
| --- | --- | --- | --- | --- | --- |
| Preferred Magnitude | Intercept | -0.23 | -0.37 - -0.12 | -3.64 | 0.004 |
|  | Day | 0.06 | -0.13 – 0.27 | 0.62 | 0.74 |
|  | Cohort | 0.08 | -0.14 – 0.34 | 0.61 | 0.74 |
|  | Day x Cohort | -0.07 | -0.5 – 0.32 | -0.34 | 0.85 |


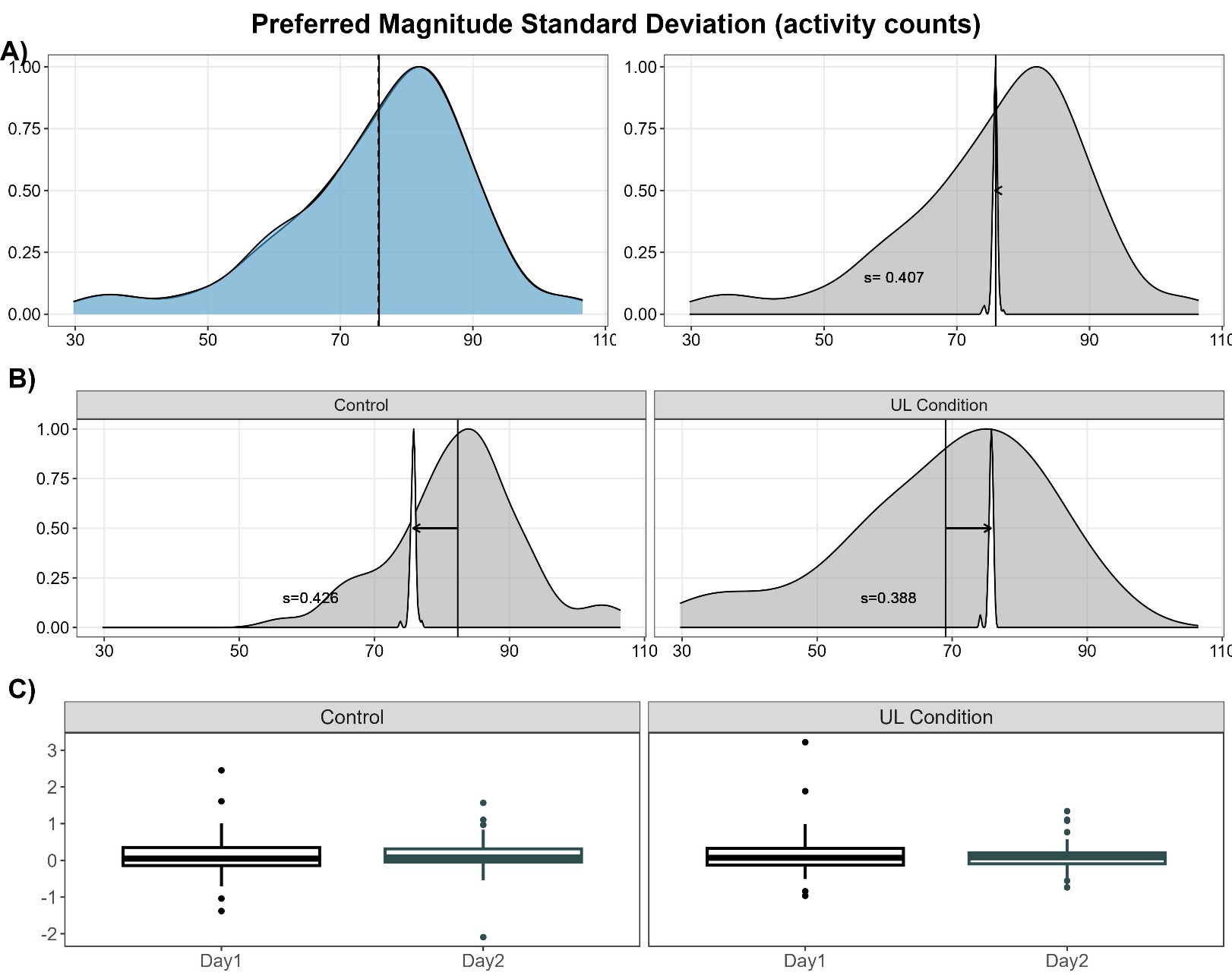


**Supplemental Figure 6. Effect of Sleep on Preferred Magnitude Standard Deviation.** Preferred magnitude standard deviation is the standard deviation of the magnitude of accelerations of the preferred limb.

Supplemental Table 7. Linear Mixed Effects Regression Results for Preferred Magnitude Standard Deviation

| **Sensor Variable** | **Model Parameter** | **Estimate** | **95% Confidence Interval** | **T-Value** | **FDR Adj. P-Value** |
| --- | --- | --- | --- | --- | --- |
| Preferred Magnitude Standard Deviation | Intercept | 0.12 | 0.05 – 0.18 | 3.31 | 0.01 |
|  | Day | -0.02 | -0.11 – 0.08 | -0.40 | 0.82 |
|  | Cohort | -0.005 | -0.13 – 0.14 | -0.07 | 0.95 |
|  | Day x Cohort | -0.09 | -0.3 – 0.1 | -0.88 | 0.69 |


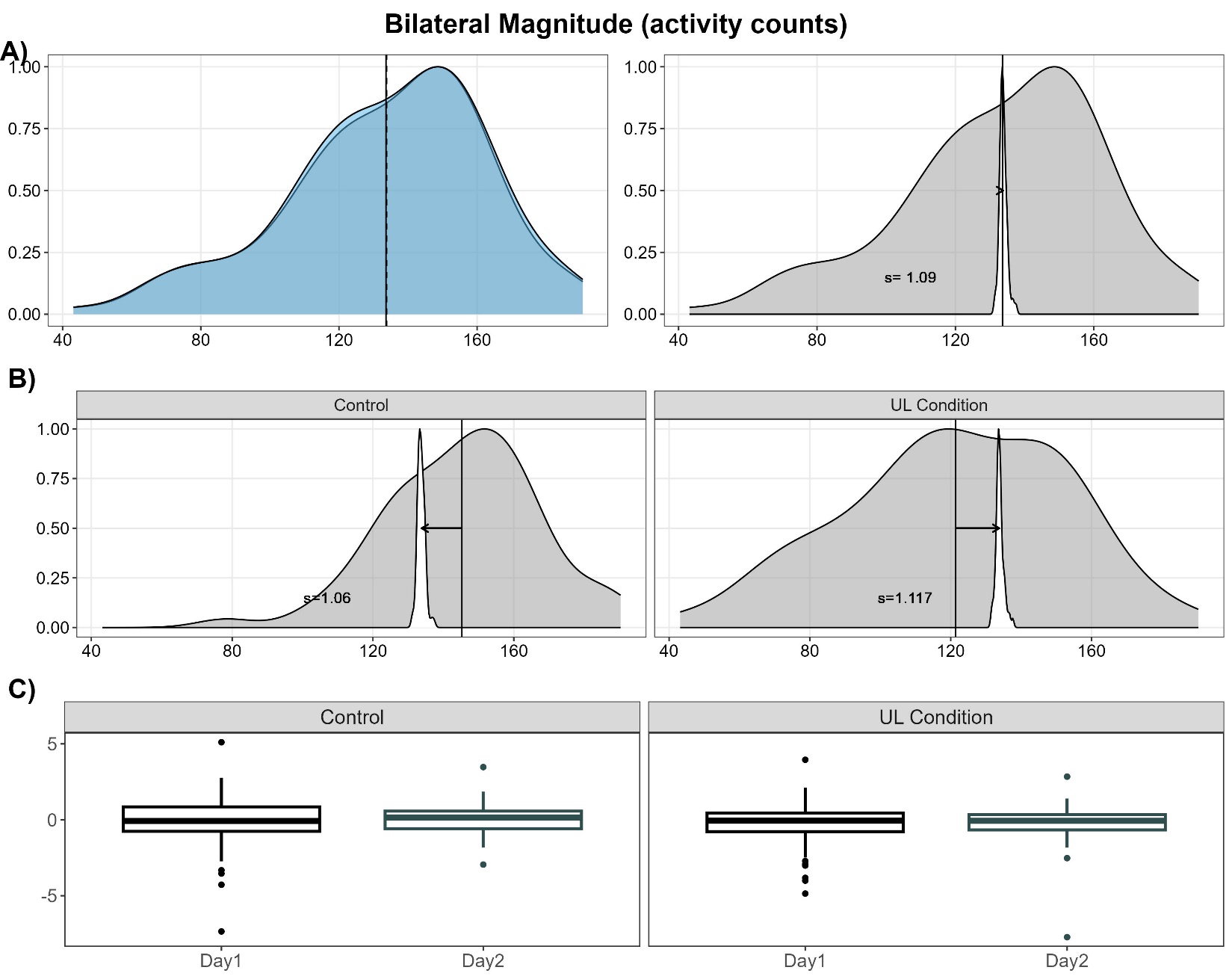


**Supplemental Figure 7. Effect of Sleep on Bilateral Magnitude.** Bilateral magnitude is the intensity, or magnitude of accelerations of movement summed across both limbs, in activity counts.

Supplemental Table 8. Linear Mixed Effects Regression Results for Bilateral Magnitude

| **Sensor Variable** | **Model Parameter** | **Estimate** | **95% Confidence Interval** | **T-Value** | **FDR Adj. P-Value** |
| --- | --- | --- | --- | --- | --- |
| Bilateral Magnitude | Intercept | -0.19 | -0.39 – 0.01 | -1.96 | 0.22 |
|  | Day | 0.1 | -0.22 – 0.4 | 0.64 | 0.74 |
|  | Cohort | -0.23 | -0.61 – 0.14 | -1.18 | 0.61 |
|  | Day x Cohort | -0.05 | -0.73 – 0.54 | -0.17 | 0.94 |


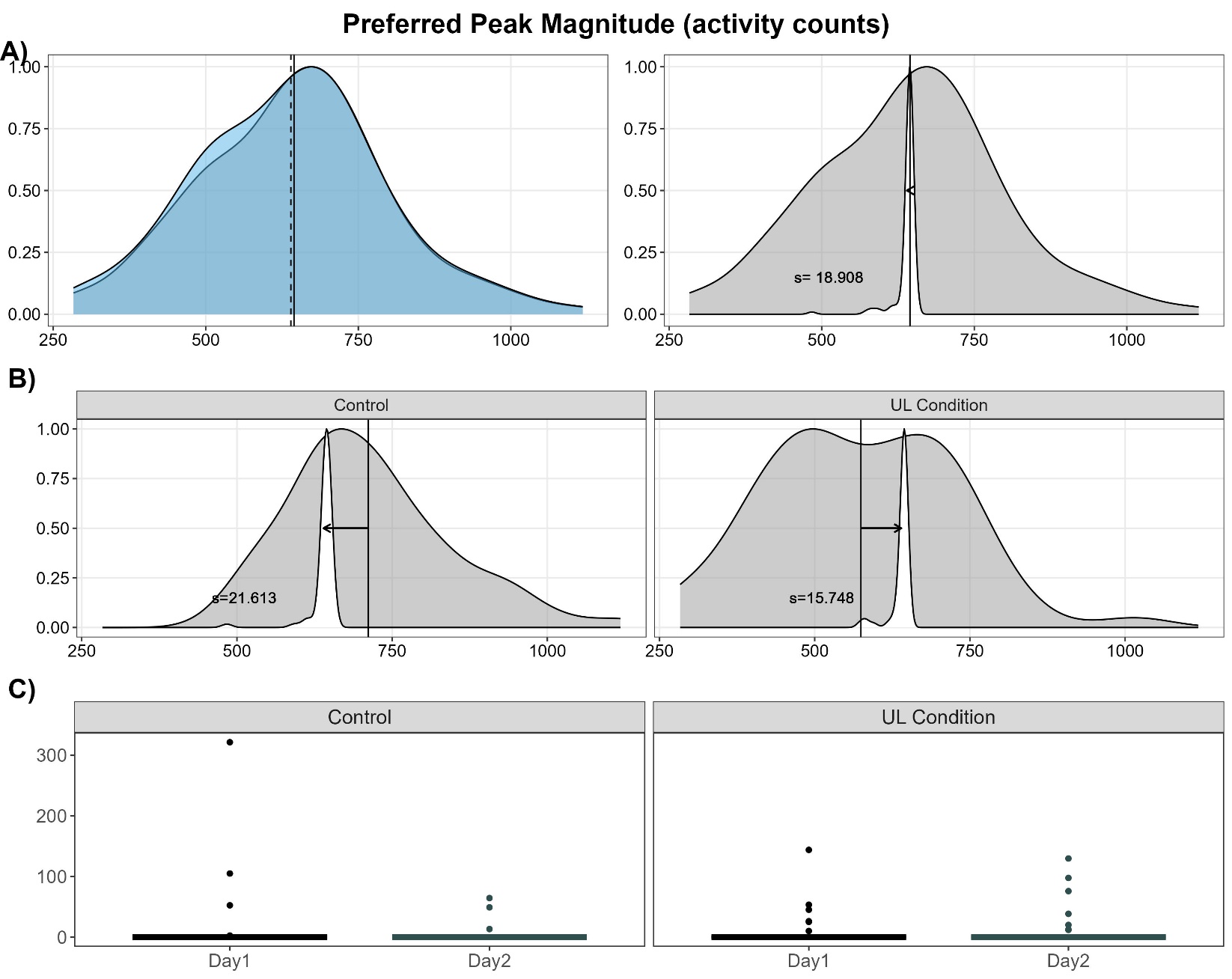


**Supplemental Figure 8. Effect of Sleep on Preferred Peak Magnitude.** Preferred peak magnitude is the highest magnitude of accelerations of the preferred limb.

Supplemental Table 9. Linear Mixed Effects Regression Results for Preferred Peak Magnitude

| **Sensor Variable** | **Model Parameter** | **Estimate** | **95% Confidence Interval** | **T-Value** | **FDR Adj. P-Value** |
| --- | --- | --- | --- | --- | --- |
| Preferred Peak Magnitude | Intercept | 5.13 | 1.92 – 8.65 | 3.07 | 0.02 |
|  | Day | -2.08 | -8.81 – 3.62 | -0.63 | 0.74 |
|  | Cohort | 0.88 | -5.52 – 7.24 | 0.26 | 0.90 |
|  | Day x Cohort | 6.81 | -7.13 – 20.93 | 1.03 | 0.61 |


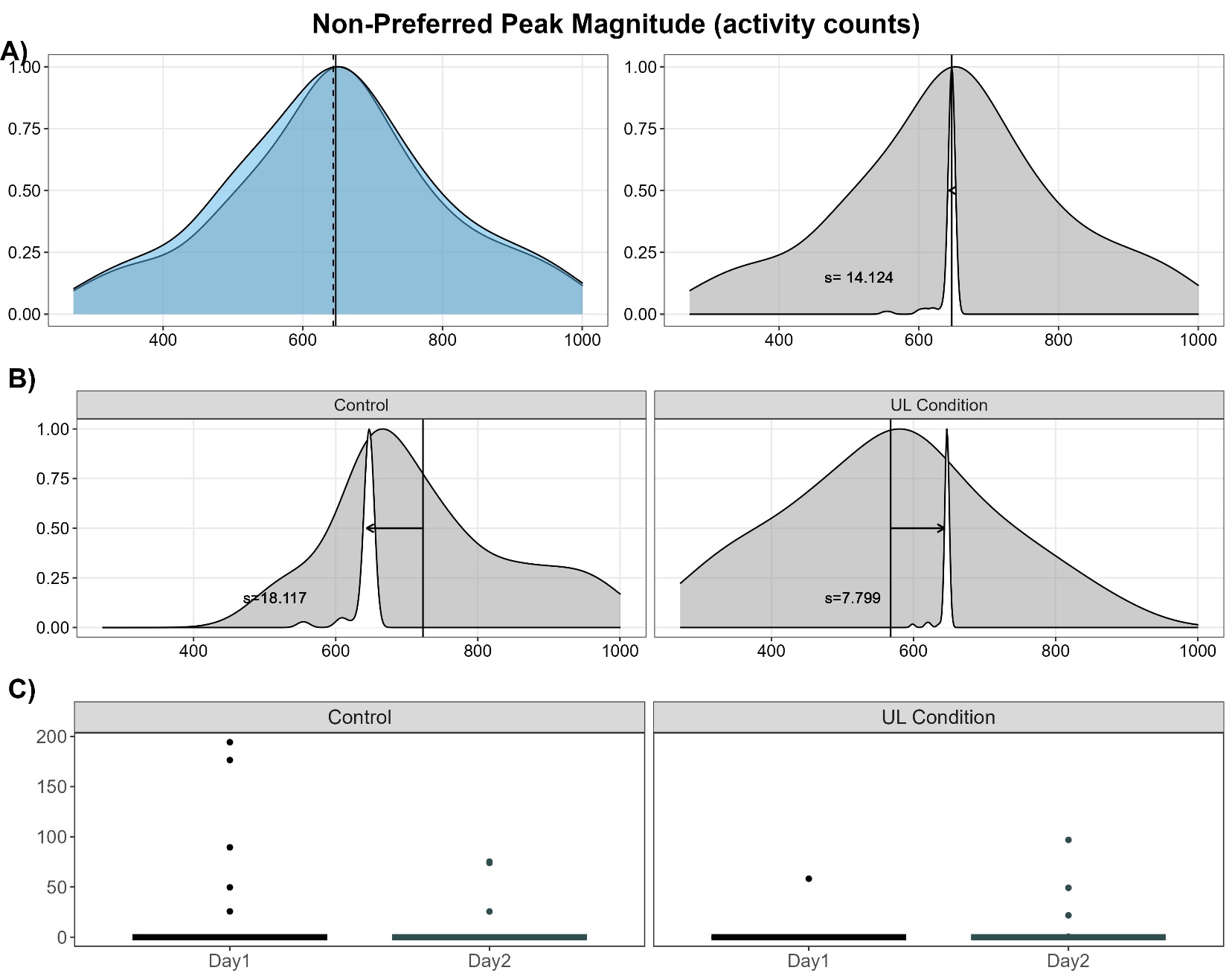


**Supplemental Figure 9. Effect of Sleep on Non-Preferred Peak Magnitude.** Non-preferred peak magnitude is the highest magnitude of accelerations of the non-preferred limb.

Supplemental Table 10. Linear Mixed Effects Regression Results for Non-Preferred Peak Magnitude

| **Sensor Variable** | **Model Parameter** | **Estimate** | **95% Confidence Interval** | **T-Value** | **FDR Adj. P-Value** |
| --- | --- | --- | --- | --- | --- |
| Non-Preferred Peak Magnitude | Intercept | 3.64 | 1.06– 6.51 | 2.90 | 0.03 |
|  | Day | -1.89 | -7.1 – 2.6 | -0.75 | 0.72 |
|  | Cohort | -3.64 | -8.34 – 0.92 | -1.45 | 0.44 |
|  | Day x Cohort | 7.33 | -2.07 – 16.68 | 1.46 | 0.44 |


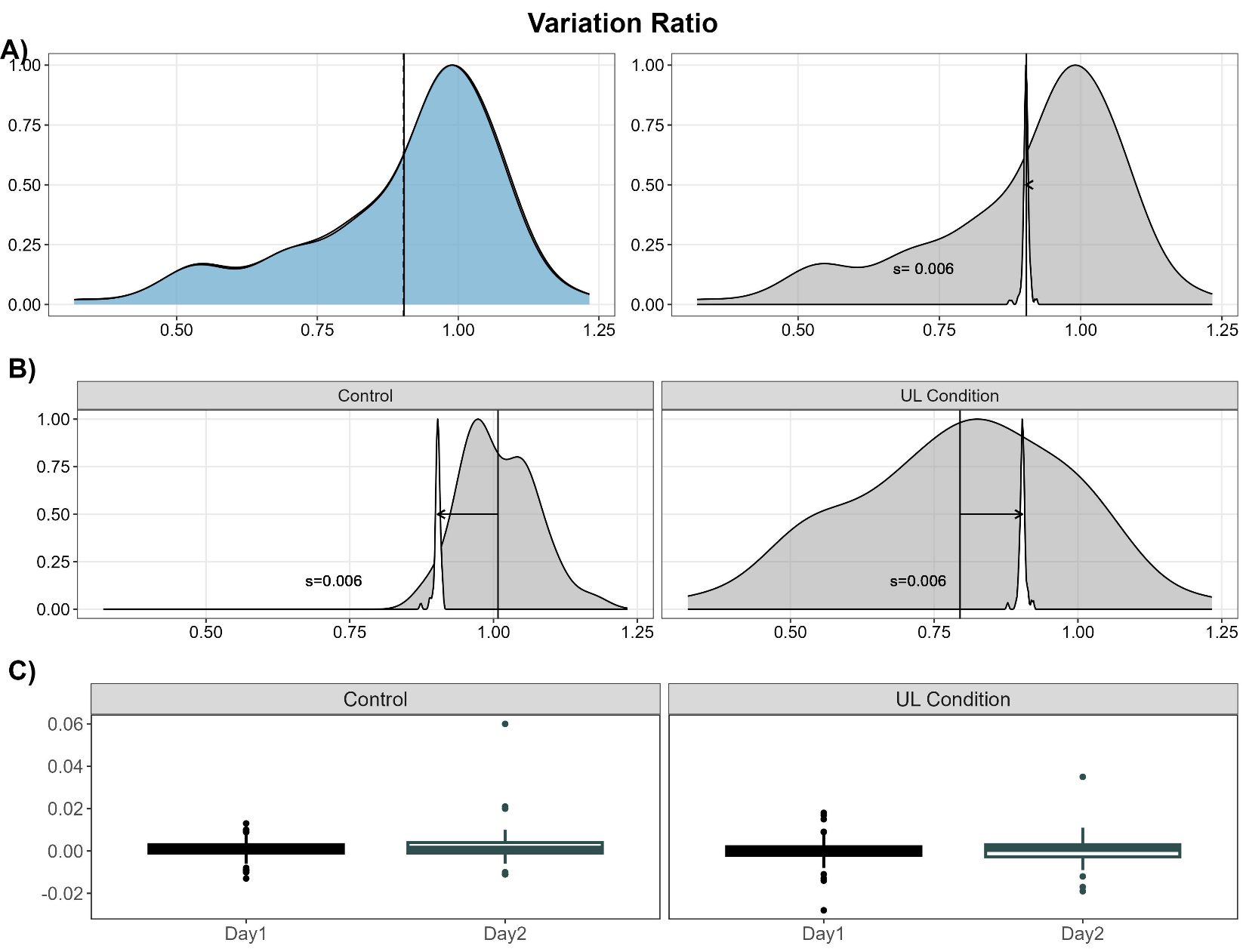


**Supplemental Figure 10. Effect of Sleep on Variation Ratio.** The variation ratio is the ratio of the standard deviation of the non-preferred limb acceleration magnitude relative to the preferred limb.

Supplemental Table 11. Linear Mixed Effects Regression Results for Variation Ratio

| **Sensor Variable** | **Model Parameter** | **Estimate** | **95% Confidence Interval** | **T-Value** | **FDR Adj. P-Value** |
| --- | --- | --- | --- | --- | --- |
| Variation Ratio | Intercept | 0.001 | -0.0001 – 0.002 | 1.65 | 0.38 |
|  | Day | 0.001 | -0.001 – 0.002 | 1.15 | 0.61 |
|  | Cohort | -0.001 | -0.003 – 0.001 | -1.05 | 0.61 |
|  | Day x Cohort | -0.002 | -0.005 – 0.001 | -1.06 | 0.61 |


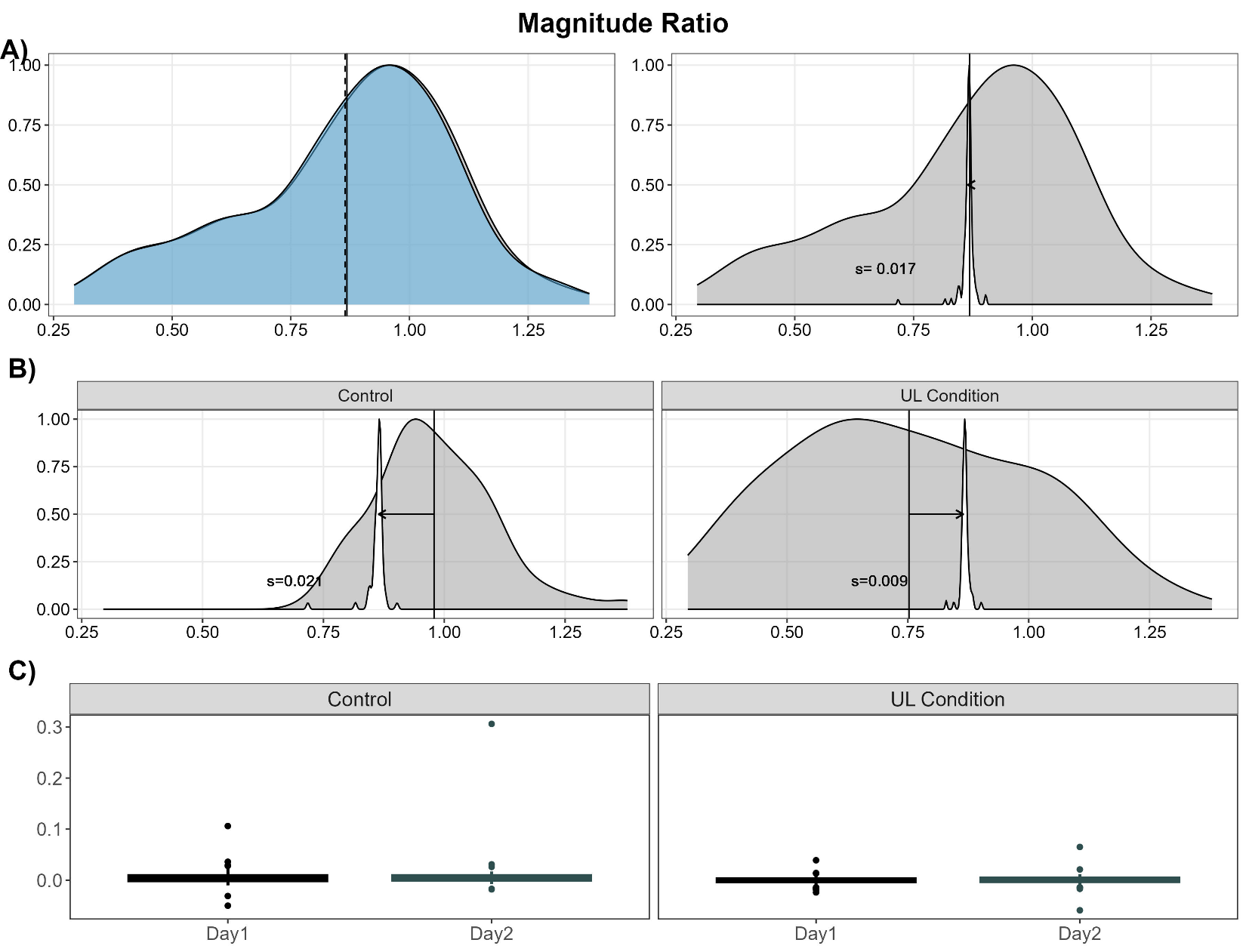


**Supplemental Figure 11. Effect of Sleep on Magnitude Ratio.** The magnitude ratio is the ratio of the magnitude of accelerations the non-preferred limb relative to the preferred limb.

Supplemental Table 12. Linear Mixed Effects Regression Results for Magnitude Ratio

| **Sensor Variable** | **Model Parameter** | **Estimate** | **95% Confidence Interval** | **T-Value** | **FDR Adj. P-Value** |
| --- | --- | --- | --- | --- | --- |
| Magnitude Ratio | Intercept | 0.004 | 0.001 – 0.01 | 2.68 | 0.05 |
|  | Day | 0.002 | -0.004 – 0.01 | 0.71 | 0.74 |
|  | Cohort | -0.01 | -0.01 - -0.002 | -2.52 | 0.07 |
|  | Day x Cohort | -0.003 | -0.01 – 0.01 | -0.60 | 0.74 |


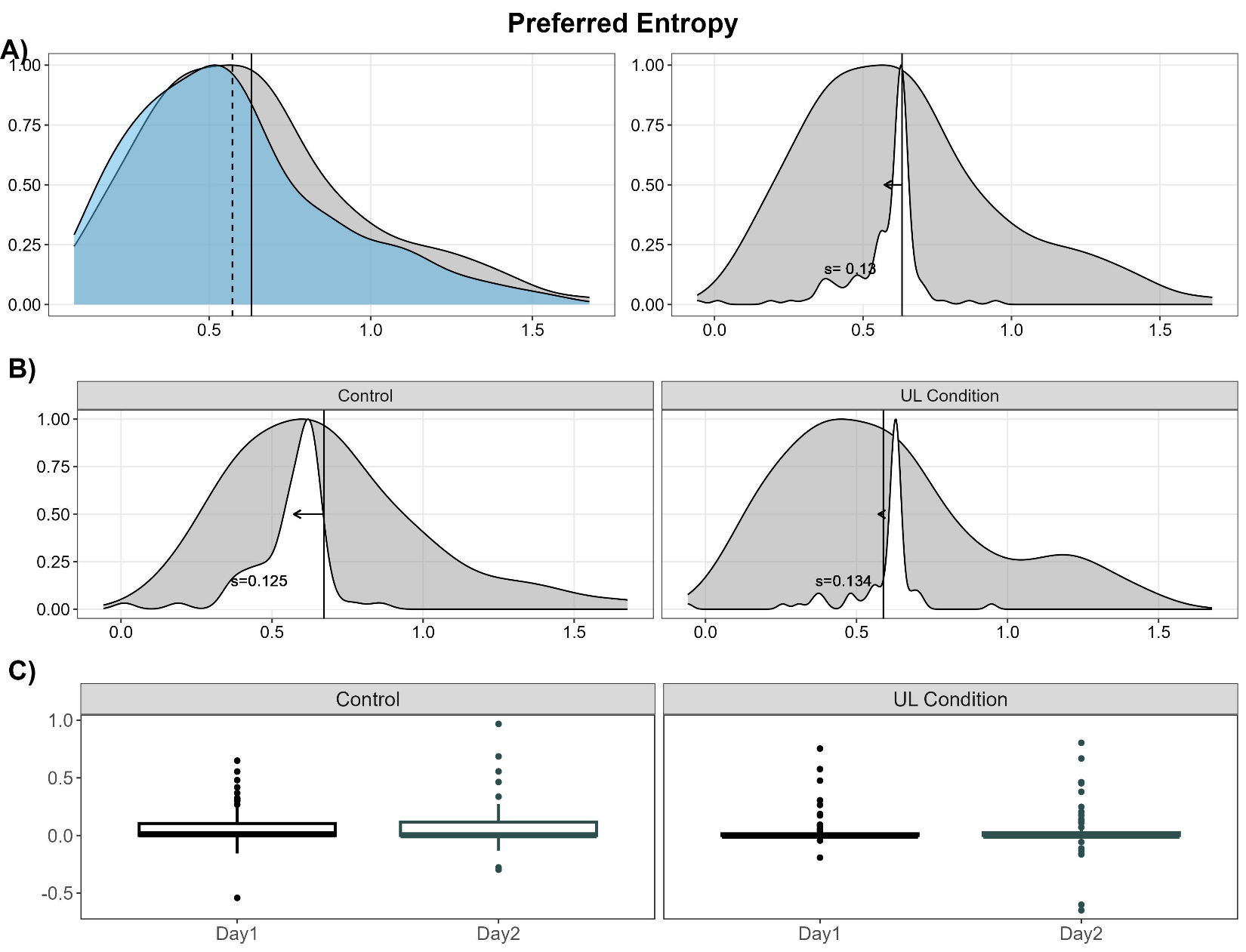


**Supplemental Figure 12. Effect of Sleep on Preferred Entropy.** Preferred entropy is a measure of the time series variability from the accelerations of the preferred limb during the hour of maximum activity. Higher values indicate a more random signal.

Supplemental Table 13. Linear Mixed Effects Regression Results for Preferred Entropy

| **Sensor Variable** | **Model Parameter** | **Estimate** | **95% Confidence Interval** | **T-Value** | **FDR Adj. P-Value** |
| --- | --- | --- | --- | --- | --- |
| Preferred Entropy | Intercept | 0.06 | 0.03 – 0.08 | 5.14 | <0.001 |
|  | Day | -0.005 | -0.05 – 0.04 | -0.21 | 0.92 |
|  | Cohort | -0.03 | -0.07 – 0.02 | -1.13 | 0.61 |
|  | Day x Cohort | -0.004 | -0.10 – 0.09 | -0.09 | 0.94 |


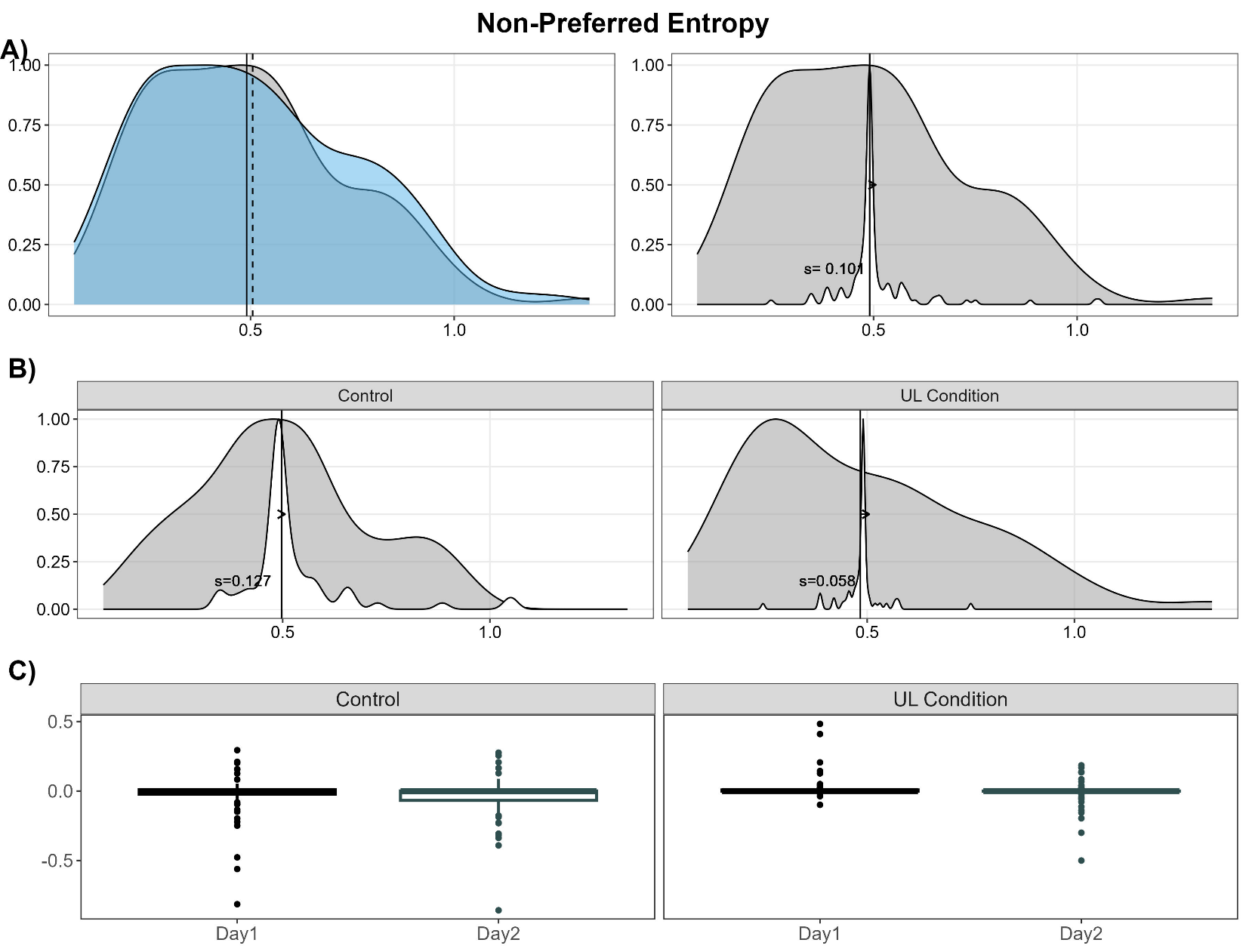


**Supplemental Figure 13. Effect of Sleep on Non-Preferred Entropy.** Non-preferred entropy is a measure of the time series variability from the accelerations of the non-preferred limb during the hour of maximum activity. Higher values indicate a more random signal.

Supplemental Table 14. Linear Mixed Effects Regression Results for Non-Preferred Entropy

| **Sensor Variable** | **Model Parameter** | **Estimate** | **95% Confidence Interval** | **T-Value** | **FDR Adj. P-Value** |
| --- | --- | --- | --- | --- | --- |
| Non-Preferred Entropy | Intercept | -0.01 | -0.03 – 0.004 | -1.62 | 0.38 |
|  | Day | -0.02 | -0.05 – 0.01 | -1.55 | 0.42 |
|  | Cohort | 0.04 | 0.01 – 0.07 | 2.29 | 0.11 |
|  | Day x Cohort | -0.03 | -0.09 – 0.03 | -0.83 | 0.71 |


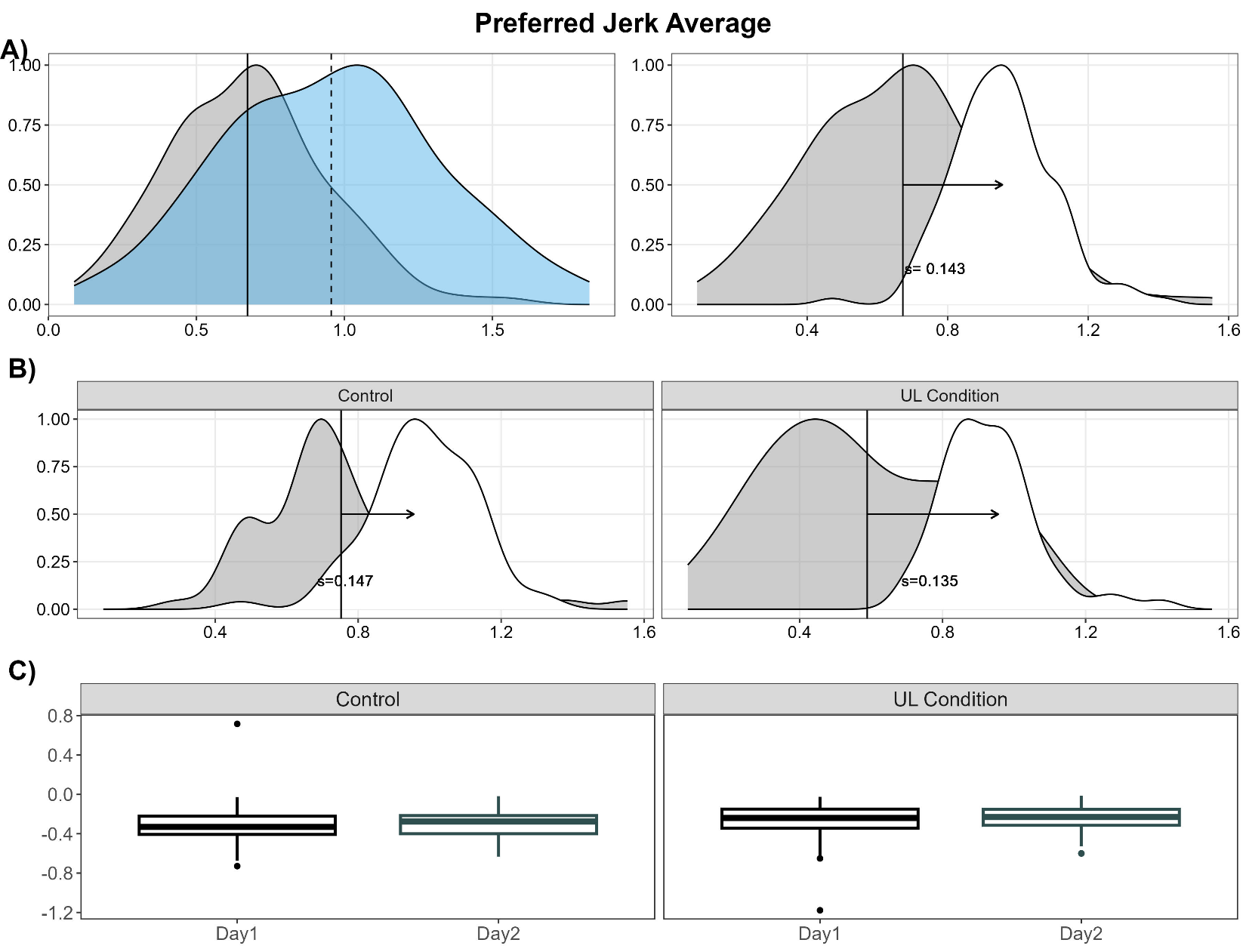


**Supplemental Figure 14. Effect of Sleep on Preferred Jerk Average.** The average jerk of the preferred limb. Higher values indicate less smooth movement.

Supplemental Table 15. Linear Mixed Effects Regression Results for Preferred Jerk Average

| **Sensor Variable** | **Model Parameter** | **Estimate** | **95% Confidence Interval** | **T-Value** | **FDR Adj. P-Value** |
| --- | --- | --- | --- | --- | --- |
| Preferred Jerk Average | Intercept | -0.28 | -0.03 – 0.004 | -22.71 | <0.001 |
|  | Day | 0.01 | -0.05 – 0.01 | 0.61 | 0.74 |
|  | Cohort | 0.05 | 0.01 – 0.07 | 2.04 | 0.19 |
|  | Day x Cohort | 0.01 | -0.09 – 0.03 | 0.42 | 0.81 |


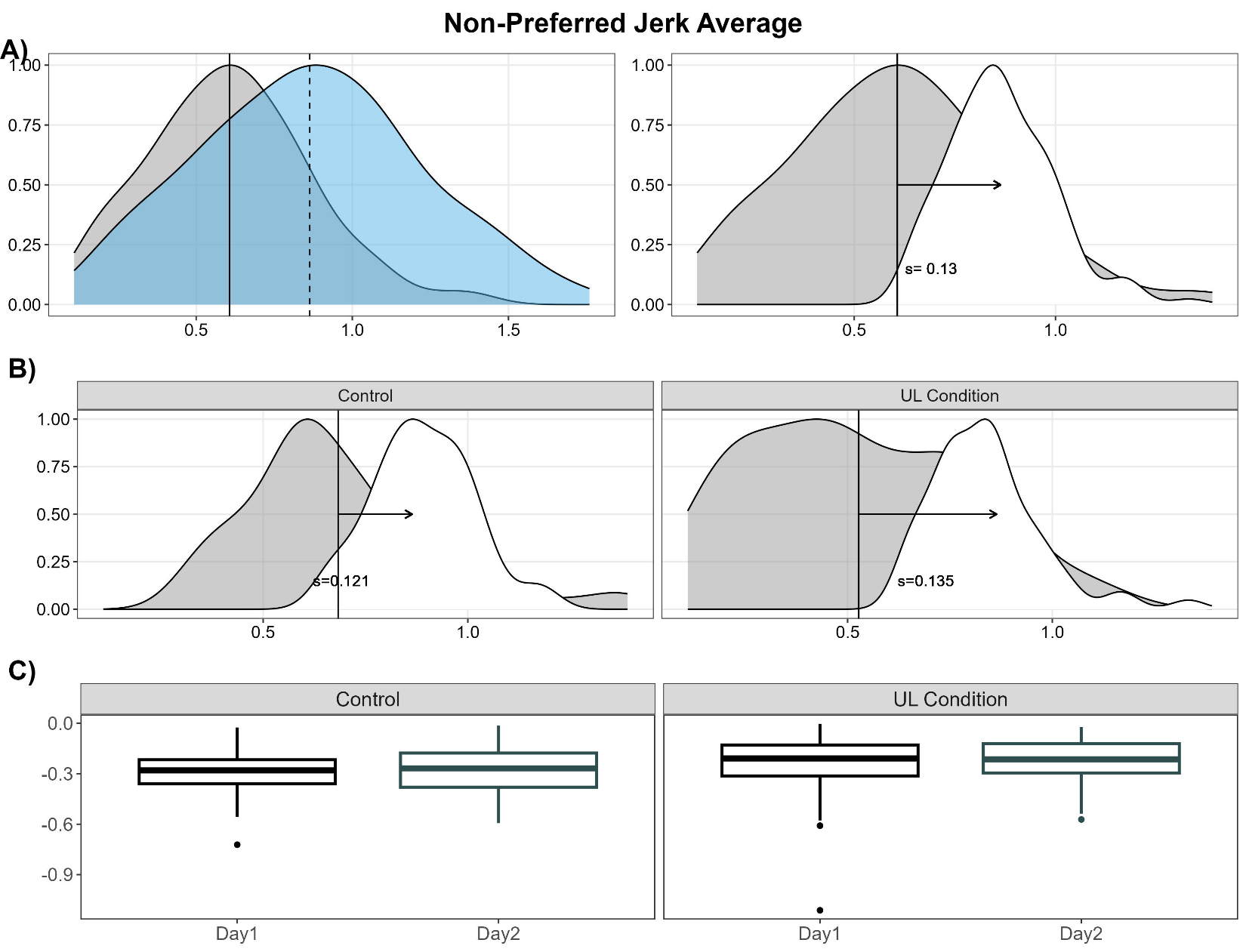


**Supplemental Figure 15. Effect of Sleep on Non-Preferred Jerk Average.** Non-preferred jerk average is the average jerk of the non-preferred limb. Higher values indicate less smooth movement.

Supplemental Table 16. Linear Mixed Effects Regression Results for Non-Preferred Jerk Average

| **Sensor Variable** | **Model Parameter** | **Estimate** | **95% Confidence Interval** | **T-Value** | **FDR Adj. P-Value** |
| --- | --- | --- | --- | --- | --- |
| Non-Preferred Jerk Average | Intercept | -0.26 | -0.28 - -0.24 | -22.73 | <0.001 |
|  | Day | 0.01 | -0.01 – 0.04 | 1.27 | 0.57 |
|  | Cohort | 0.05 | 0.01 – 0.10 | 2.40 | 0.09 |
|  | Day x Cohort | -0.003 | -0.05 – 0.04 | -0.14 | 0.94 |


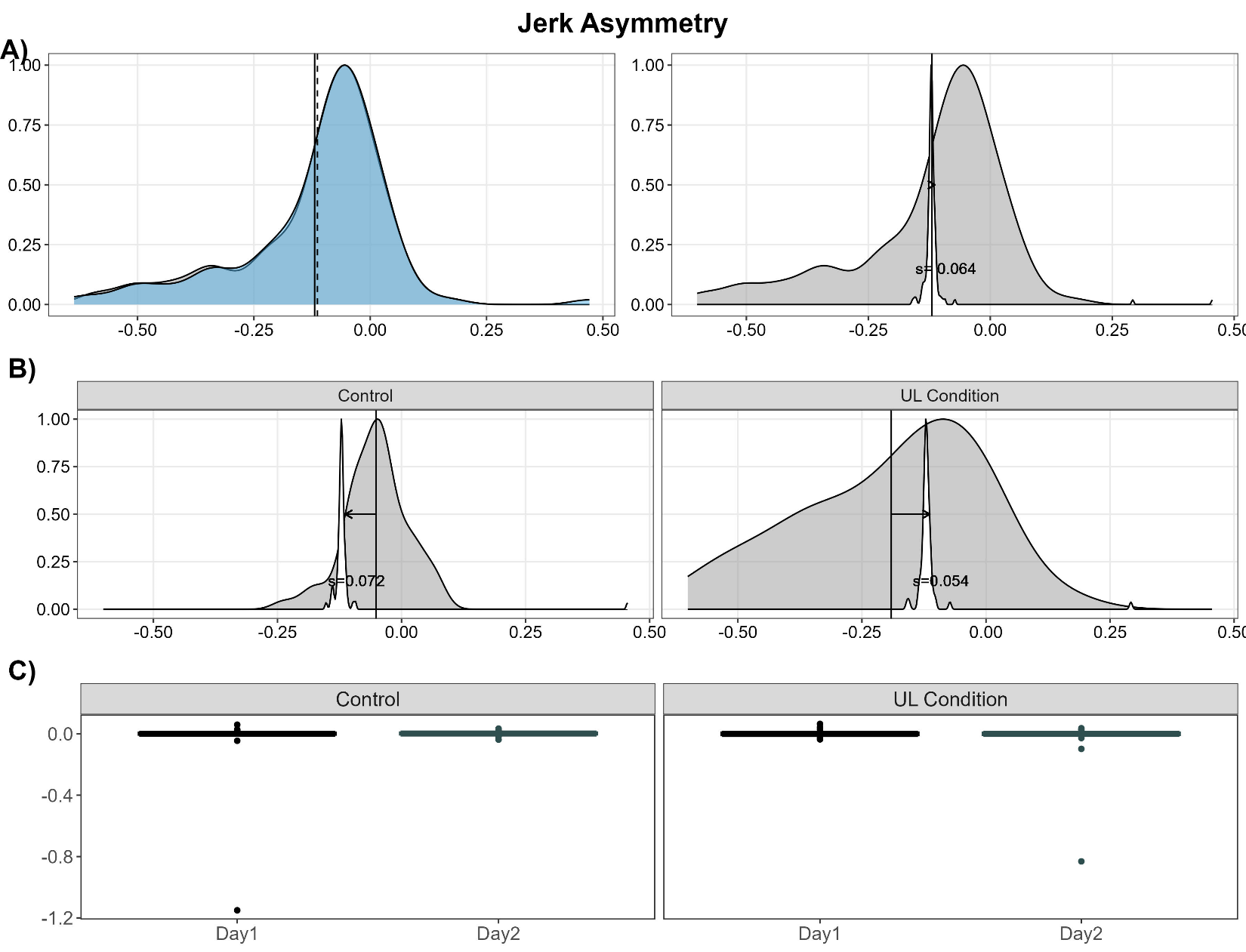


**Supplemental Figure 16. Effect of Sleep on Jerk Asymmetry.** Jerk asymmetry is the ratio of the average jerk magnitude between the non-preferred and the preferred limb. Higher jerk represents less smooth movement. A value of 0 represents similar smoothness of movement on both limbs.

Supplemental Table 17. Linear Mixed Effects Regression Results for Jerk Asymmetry

| **Sensor Variable** | **Model Parameter** | **Estimate** | **95% Confidence Interval** | **T-Value** | **FDR Adj. P-Value** |
| --- | --- | --- | --- | --- | --- |
| Jerk Asymmetry | Intercept | -0.01 | -0.02 – 0.01 | -1.08 | 0.61 |
|  | Day | 0.002 | -0.02 – 0.02 | 0.15 | 0.94 |
|  | Cohort | 0.001 | -0.02 – 0.02 | 0.10 | 0.94 |
|  | Day x Cohort | -0.03 | -0.08 – 0.01 | -1.46 | 0.44 |


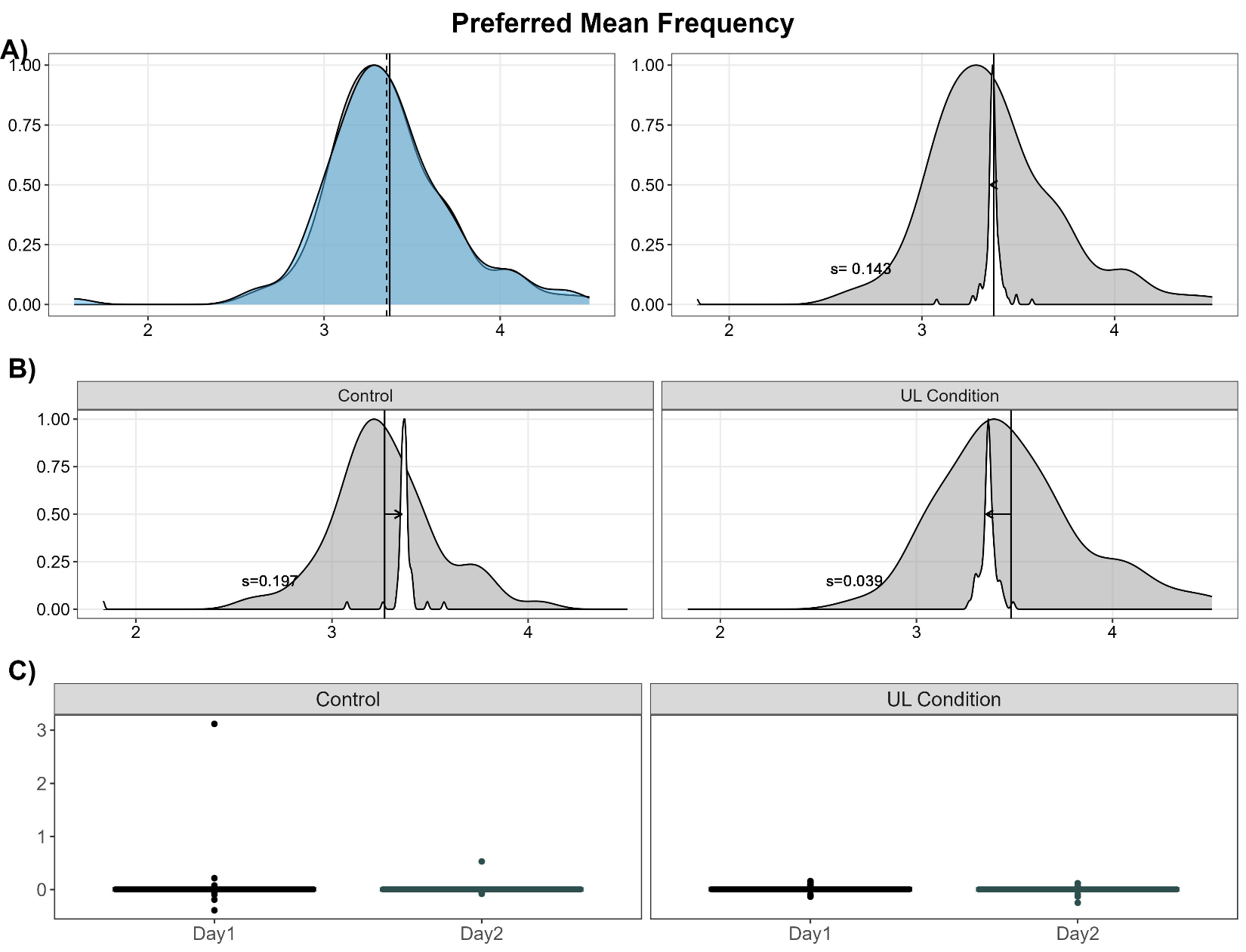


**Supplemental Figure 17. Effect of Sleep on Preferred Mean Frequency.** Preferred mean frequency is the weighted mean of the component frequencies from the acceleration time series from the preferred limb.

Supplemental Table 18. Linear Mixed Effects Regression Results for Preferred Mean Frequency

| **Sensor Variable** | **Model Parameter** | **Estimate** | **95% Confidence Interval** | **T-Value** | **FDR Adj. P-Value** |
| --- | --- | --- | --- | --- | --- |
| Preferred Mean Frequency | Intercept | 0.02 | -0.005 – 0.04 | 1.35 | 0.51 |
|  | Day | -0.03 | -0.08 – 0.02 | -1.0 | 0.61 |
|  | Cohort | -0.02 | -0.07 – 0.03 | -0.86 | 0.70 |
|  | Day x Cohort | 0.03 | -0.09 – 0.13 | 0.51 | 0.78 |


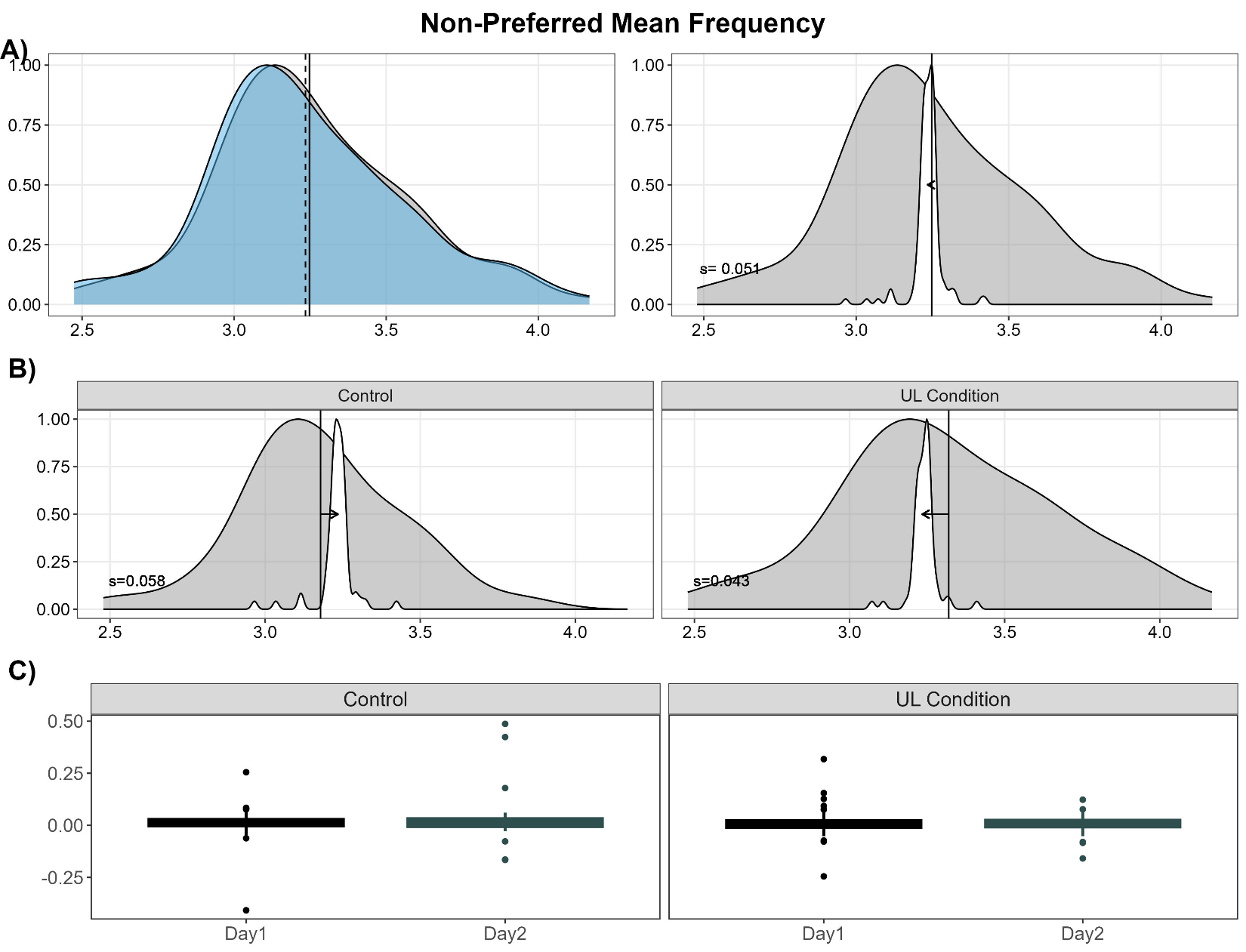


**Supplemental Figure 18. Effect of Sleep on Non-Preferred Mean Frequency.** Non-preferred mean frequency is the weighted mean of the component frequencies from the acceleration time series from the non-preferred limb.

Supplemental Table 19. Linear Mixed Effects Regression Results for Non-Preferred Mean Frequency

| **Sensor Variable** | **Model Parameter** | **Estimate** | **95% Confidence Interval** | **T-Value** | **FDR Adj. P-Value** |
| --- | --- | --- | --- | --- | --- |
| Non-Preferred Mean Frequency | Intercept | 0.01 | 0.003 – 0.02 | 2.88 | 0.03 |
|  | Day | 0.003 | -0.01 – 0.02 | 0.33 | 0.86 |
|  | Cohort | -0.01 | -0.03 – 0.01 | -1.02 | 0.61 |
|  | Day x Cohort | -0.02 | -0.05 – 0.02 | -1.00 | 0.61 |


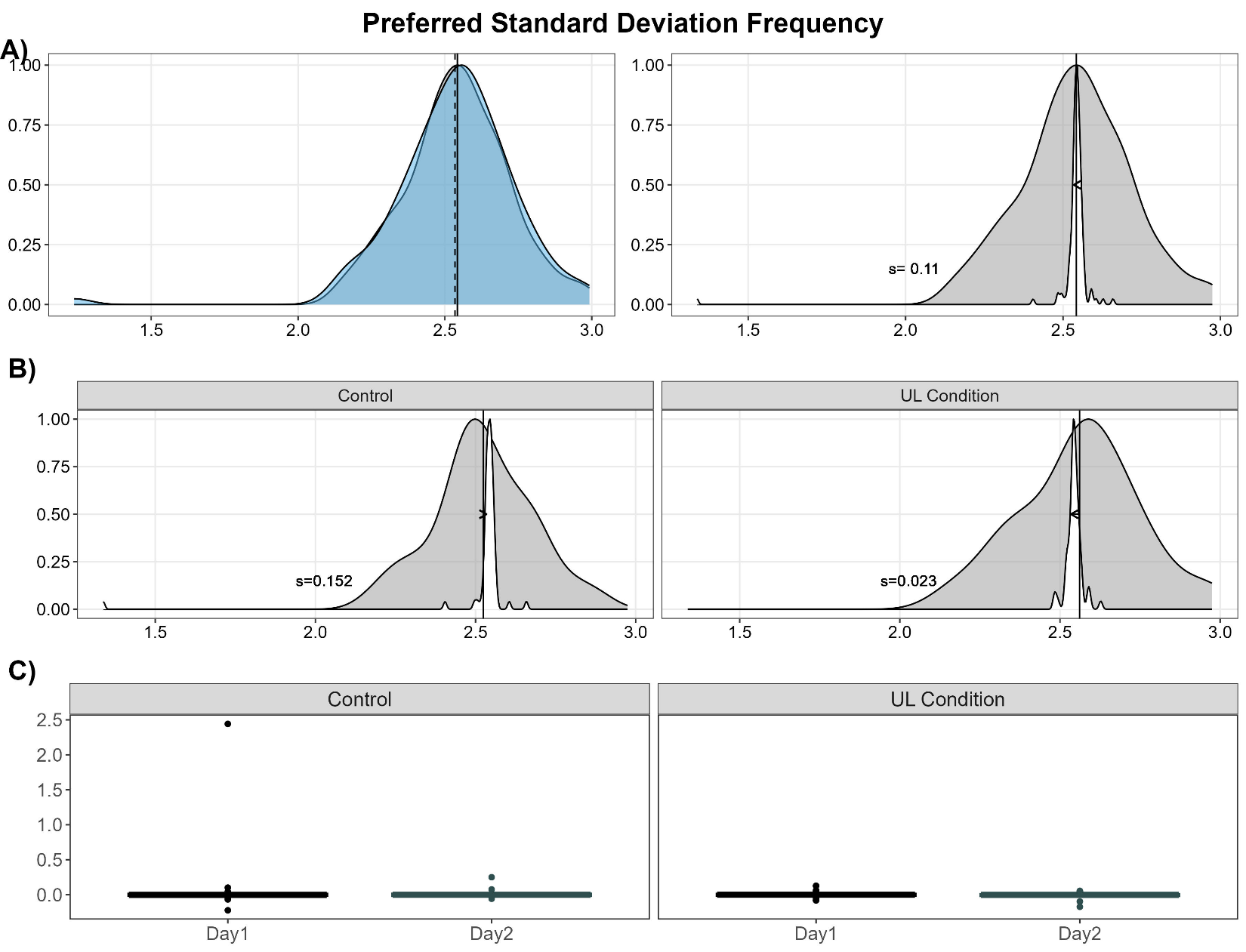


**Supplemental Figure 19. Effect of Sleep on Preferred Standard Deviation Frequency.** Preferred standard deviation frequency is the weighted standard deviation of the component frequencies from the acceleration time series from the preferred limb.

Supplemental Table 20. Linear Mixed Effects Regression Results for Preferred Standard Deviation Frequency

| **Sensor Variable** | **Model Parameter** | **Estimate** | **95% Confidence Interval** | **T-Value** | **FDR Adj. P-Value** |
| --- | --- | --- | --- | --- | --- |
| Preferred Standard Deviation Frequency | Intercept | 0.01 | -0.01 – 0.02 | 0.80 | 0.72 |
|  | Day | -0.02 | -0.06 – 0.02 | -0.89 | 0.69 |
|  | Cohort | -0.02 | -0.06 – 0.02 | -1.01 | 0.61 |
|  | Day x Cohort | 0.02 | -0.06 – 0.10 | 0.59 | 0.74 |


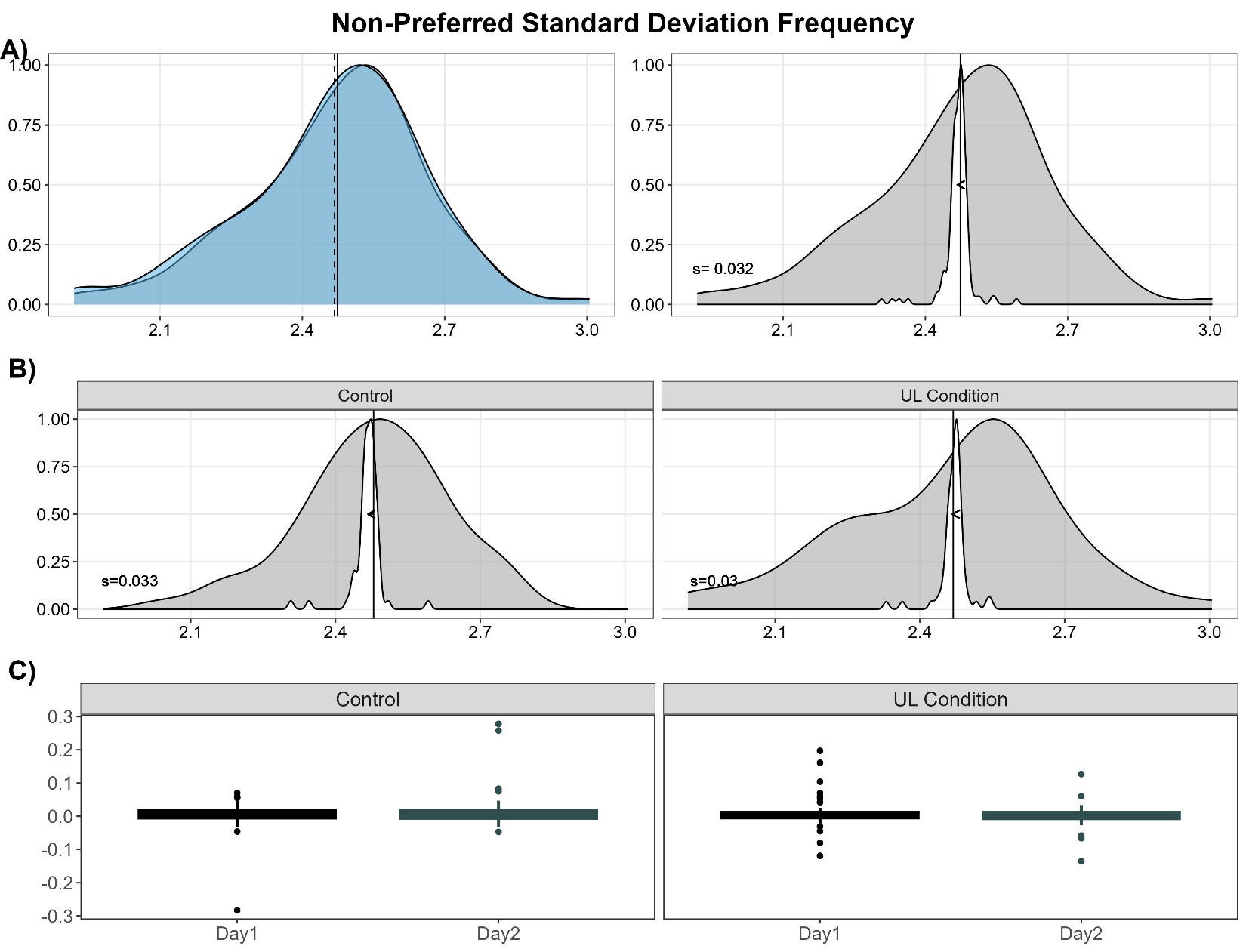


**Supplemental Figure 20. Effect of Sleep on Non-Preferred Standard Deviation Frequency.** Non-preferred standard deviation frequency is the weighted standard deviation of the component frequencies from the acceleration time series from the non-preferred limb.

Supplemental Table 21. Linear Mixed Effects Regression Results for Non-Preferred Standard Deviation Frequency

| **Sensor Variable** | **Model Parameter** | **Estimate** | **95% Confidence Interval** | **T-Value** | **FDR Adj. P-Value** |
| --- | --- | --- | --- | --- | --- |
| Non-Preferred Standard Deviation Frequency | Intercept | 0.01 | 0.001 – 0.01 | 2.30 | 0.11 |
|  | Day | 0.002 | -0.01 – 0.01 | 0.50 | 0.78 |
|  | Cohort | -0.004 | -0.02 – 0.01 | -0.67 | 0.74 |
|  | Day x Cohort | -0.02 | -0.04 – 0.003 | -1.76 | 0.32 |
